# The Length of the Expressed 3′ UTR Is an Intermediate Molecular Phenotype Linking Genetic Variants to Complex Diseases

**DOI:** 10.3389/fgene.2019.00714

**Published:** 2019-08-16

**Authors:** Elisa Mariella, Federico Marotta, Elena Grassi, Stefano Gilotto, Paolo Provero

**Affiliations:** ^1^Department of Molecular Biotechnology and Health Sciences, University of Turin, Turin, Italy; ^2^Center for Tranlational Genomics and Bioinformatics, San Raffaele Scientific Institute, Milan, Italy

**Keywords:** human genetic variants, alternative polyadenylation, quantitative trait loci (QTL), whole-genome sequencing (WGS), RNA sequencing (RNA-Seq), genome-wide association studies (GWAS)

## Abstract

In the last decades, genome-wide association studies (GWAS) have uncovered tens of thousands of associations between common genetic variants and complex diseases. However, these statistical associations can rarely be interpreted functionally and mechanistically. As the majority of the disease-associated variants are located far from coding sequences, even the relevant gene is often unclear. A way to gain insight into the relevant mechanisms is to study the genetic determinants of intermediate molecular phenotypes, such as gene expression and transcript structure. We propose a computational strategy to discover genetic variants affecting the relative expression of alternative 3′ untranslated region (UTR) isoforms, generated through alternative polyadenylation, a widespread posttranscriptional regulatory mechanism known to have relevant functional consequences. When applied to a large dataset in which whole genome and RNA sequencing data are available for 373 European individuals, 2,530 genes with alternative polyadenylation quantitative trait loci (apaQTL) were identified. We analyze and discuss possible mechanisms of action of these variants, and we show that they are significantly enriched in GWAS hits, in particular those concerning immune-related and neurological disorders. Our results point to an important role for genetically determined alternative polyadenylation in affecting predisposition to complex diseases, and suggest new ways to extract functional information from GWAS data.

## Introduction

Understanding the relationship between human genotypes and phenotypes is one of the central goals of biomedical research. The first sequencing of the human genome (International Human Genome Sequencing Consortium, 2001; Venter et al., 2001) and the following large-scale investigations of genetic differences between individuals by efforts such as the 1000 Genome Project Consortium (The 1000 Genomes Project Consortium, 2015) provided the foundation for the study of human genetics at the genome-wide level.

Genome-wide association studies (GWAS) examine common genetic variants to identify associations with complex traits, including common diseases. Long lists of genetic associations with disparate traits have been obtained, but their functional interpretation is far from being straightforward (Visscher et al., 2017). Indeed, because of linkage disequilibrium, GWAS identify genomic regions carrying multiple variants among which it is not possible to identify the causal ones without additional information. Furthermore, most loci identified in human GWAS are in noncoding regions, presumably exerting regulatory effects, but usually, we do not know the identity of the affected gene or the molecular mechanism involved.

A possible way to gain insight into the mechanisms behind GWAS associations is to investigate the effect of genetic variants on intermediate molecular phenotypes, such as gene expression (Cookson et al., 2009; Albert and Kruglyak, 2015). Expression quantitative trait loci (eQTL) are genomic regions carrying one or more genetic variants affecting gene expression. Besides their intrinsic interest in understanding the control of gene expression, eQTL studies can be exploited for the interpretation of GWAS results, helping to prioritize likely causal variants and supporting the formulation of mechanistic hypotheses about the links between genetic variants and diseases.

Recent studies have shown that genetic variants acting on the whole RNA processing cascade are at least equally common as, and largely independent from, those that affect transcriptional activity and that they can be a major driver of phenotypic variability in humans (Manning and Cooper, 2017). Therefore, it is important to identify the genetic variants associated to transcript structure, including splicing and alternative untranslated region (UTR) isoforms, besides those affecting transcriptional levels, and different approaches have been proposed to this end (Lappalainen et al., 2013; Monlong et al., 2014). From these studies, it emerges in particular that genetic variants frequently determine changes in the length of the expressed 3′ UTRs, and that these variants can be located not only within the 3′ UTR itself but also in regulatory regions outside the transcript (Lappalainen et al., 2013). In addition, genome-wide analyses specifically focused on alternative splicing have been performed (Ardlie et al., 2015; Xiong et al., 2015).

Polyadenylation is one of the posttranscriptional modifications affecting pre-mRNAs in the nucleus and involves two steps: the cleavage of the transcript and the addition of a poly(A) tail (Elkon et al., 2013; Tian and Manley, 2017). The most important regulatory elements involved are the polyadenylation signal (PAS) and other cis-elements, usually located within the 3′ UTR, but multiple and diversified regulatory mechanisms have been described (Oktaba et al., 2015; Yue et al., 2018). The PAS is recognized by the cleavage and polyadenylation specificity factor (*CPSF*) that, together with other protein complexes, induces the cleavage of the transcript in correspondence of the downstream poly(A) site. The large majority of human genes has multiple poly(A) sites so that alternative polyadenylation (APA) is a widespread phenomenon contributing to the diversification of the human transcriptome through the generation of alternative mature transcripts with different 3′ ends. Such transcripts are translated into identical proteins, but protein level, localization, and even interactions can depend on the 3′ end of the transcript (Mayr, 2018).

APA events have been grouped into classes based on the location of the alternative poly(A) site and the type of change determined by their differential usage (Elkon et al., 2013). In this work, we have taken into consideration only the simplest and most frequent mode (tandem 3′ UTR APA), in which two poly(A) sites located within the same terminal exon, one in a proximal and one in a distal position, produce transcripts that differ only in the length of the 3′ UTR. Such variation in 3′ UTR length can have an important functional impact, for example by affecting the binding of microRNAs and RNA-binding proteins and thus transcript abundance, translation, and localization. Moreover, APA regulation is strongly tissue and cell type dependent (Sandberg et al., 2008; Ji et al., 2009; Mayr and Bartel, 2009; Fu et al., 2011; Masamha et al., 2014), and several examples are known of altered APA regulation associated to human diseases (Chang et al., 2017b; Manning and Cooper, 2017).

How genetic variants influence APA has not been comprehensively investigated in a large human population yet. A recent analysis of whole-genome sequencing (WGS) data from Lappalainen et al. (2013) found hundreds of common single nucleotide polymorphisms (SNPs) causing the alteration or degradation of motifs that are similar to the canonical PAS (Ferreira et al., 2016) but did not extend the analysis to other possible mechanisms. Other studies found strong associations between genetic variants and APA regulation (Kwan et al., 2008; Thomas and Sætrom, 2012; Yoon et al., 2012; Lappalainen et al., 2013; Zhernakova et al., 2013; Monlong et al., 2014), but a systematic investigation based on a large number of samples and variants, specifically targeted to APA rather than generically to transcript structure, and unbiased in the choice of variants to examine, is not yet available.

Here, we propose a new computational strategy for the genome-wide investigation of the influence of genetic variants on the expression of alternative 3′ UTR isoforms in a large population. In particular, we analyzed WGS data paired with standard RNA-Seq data obtained in 373 European (EUR) individuals (Lappalainen et al., 2013). Statistically, our approach is analogous to methods commonly implemented in eQTL mapping analysis, and it aims to overcome the limitations illustrated above for the specific purpose of correlating variants to 3′ UTR isoforms.

A central task, preliminary to the analysis of genetic variants, is thus the quantification of the alternative 3′ UTR isoforms. Various strategies have been implemented to this end, from custom analysis pipelines for microarray data (Lembo et al., 2012), to the development of next-generation sequencing technologies specifically targeted to the 3′ end of transcripts, such as the serial analysis of gene expression (SAGE) (Ji et al., 2009) and sequencing of APA sites (SAPAs) (Fu et al., 2011), allowing also the identification of previously unannotated APA sites.

More recently, tools able to capture APA events from standard RNA-Seq data have been developed. In general, these approaches can be divided into two categories: those that exploit previous annotation of poly(A) sites (Grassi et al., 2016; Ha et al., 2018), such the ones provided by PolyA_DB2 (Lee et al., 2007) and APASdb (You et al., 2015), and those that instead try to infer their location from the data (Masamha et al., 2014). Although the latter approach potentially allows analyzing also previously unannotated sites, the former leads to higher sensitivity (Grassi et al., 2016; Ha et al., 2018) and was thus preferred in this study. Undoubtedly, approaches based on standard RNA-Seq are not as powerful and accurate as technologies that specifically sequence the 3′ ends. However, they allow studying this phenomenon in an uncomparably larger number of samples and conditions, including the recently generated large-scale transcriptomic datasets of normal individuals that we use in this work.

## Results

### Genetic Variants Affect the Relative Expression of Alternative 3′ UTR Isoforms of Thousands of Genes

**Figure 1:**
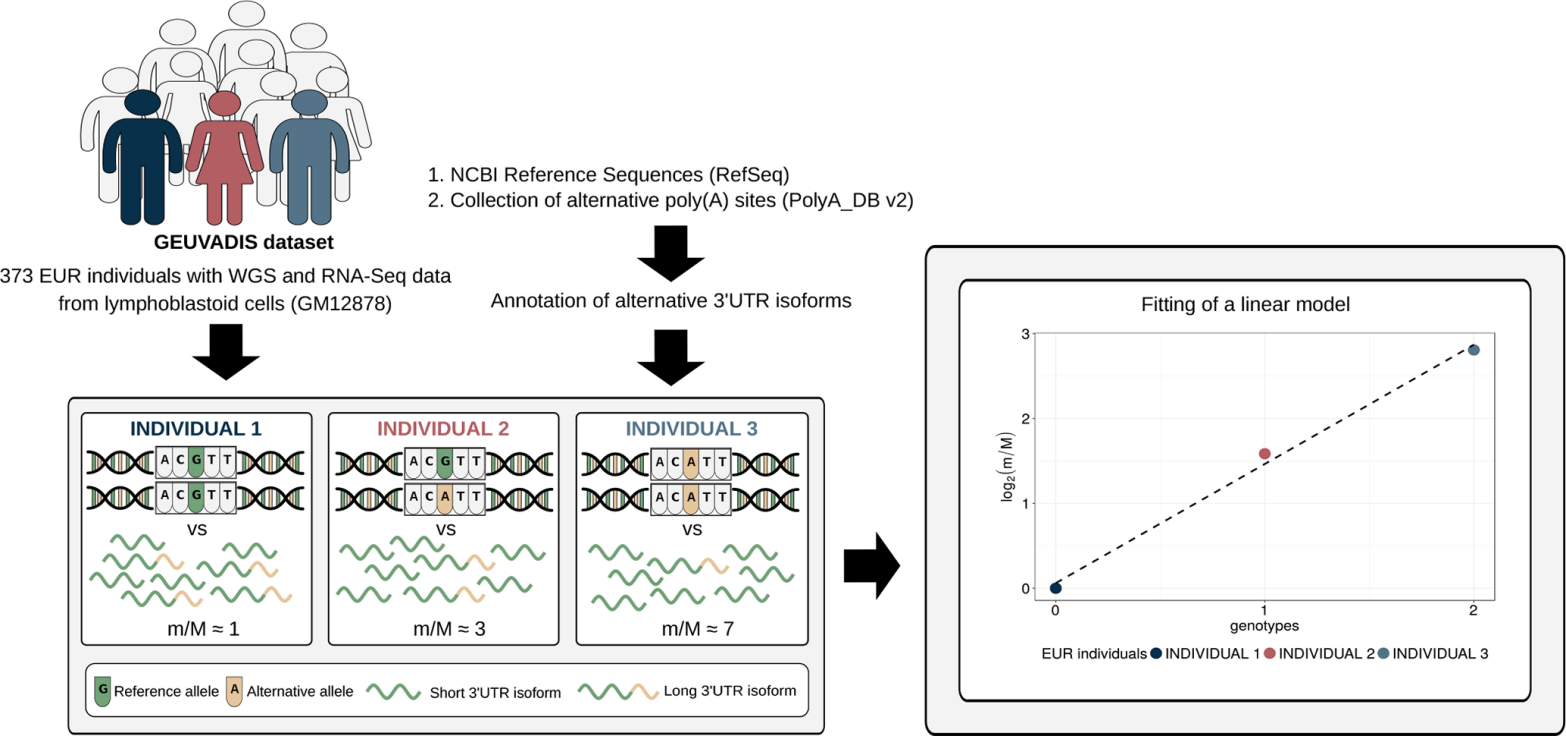
Schematic representation of the method. Genotypic data paired with RNA-Seq data from a large cohort of individuals are required to perform alternative polyadenylation quantitative trait loci (apaQTL) mapping analysis. RNA-Seq data are exploited, together with an annotation of alternative 3′ untranslated region (UTR) isoforms, to compute for each gene the *m*/*M* value that is proportional to the ratio between the expression of its short and long 3′ UTR isoforms. Then, the association between the *m*/*M* values of a gene and each nearby genetic variant is evaluated by linear regression. Genotypes are defined in the standard way: 0 means homozygous for the reference allele, 1 means heterozygous, and 2 indicates the presence of two copies of the alternative allele.

To investigate the effect of human genetic variants on the expression of alternative 3′ UTR isoforms, we developed a computational approach similar to the one commonly used for eQTL analysis (**Figure 1**). It was applied to a large dataset in which WGS data paired with RNA-Seq data are available for 373 European (EUR) individuals [GEUVADIS dataset (Lappalainen et al., 2013)]. A collection of known alternative poly(A) sites (Lee et al., 2007) was used, together with a compendium of human transcripts, to obtain an annotation of alternative 3′ UTR isoforms that was then combined with RNA-Seq data to compute, for each gene, the expression ratio between short and long isoform (*m*/*M* value) in each individual.

**Figure 2:**
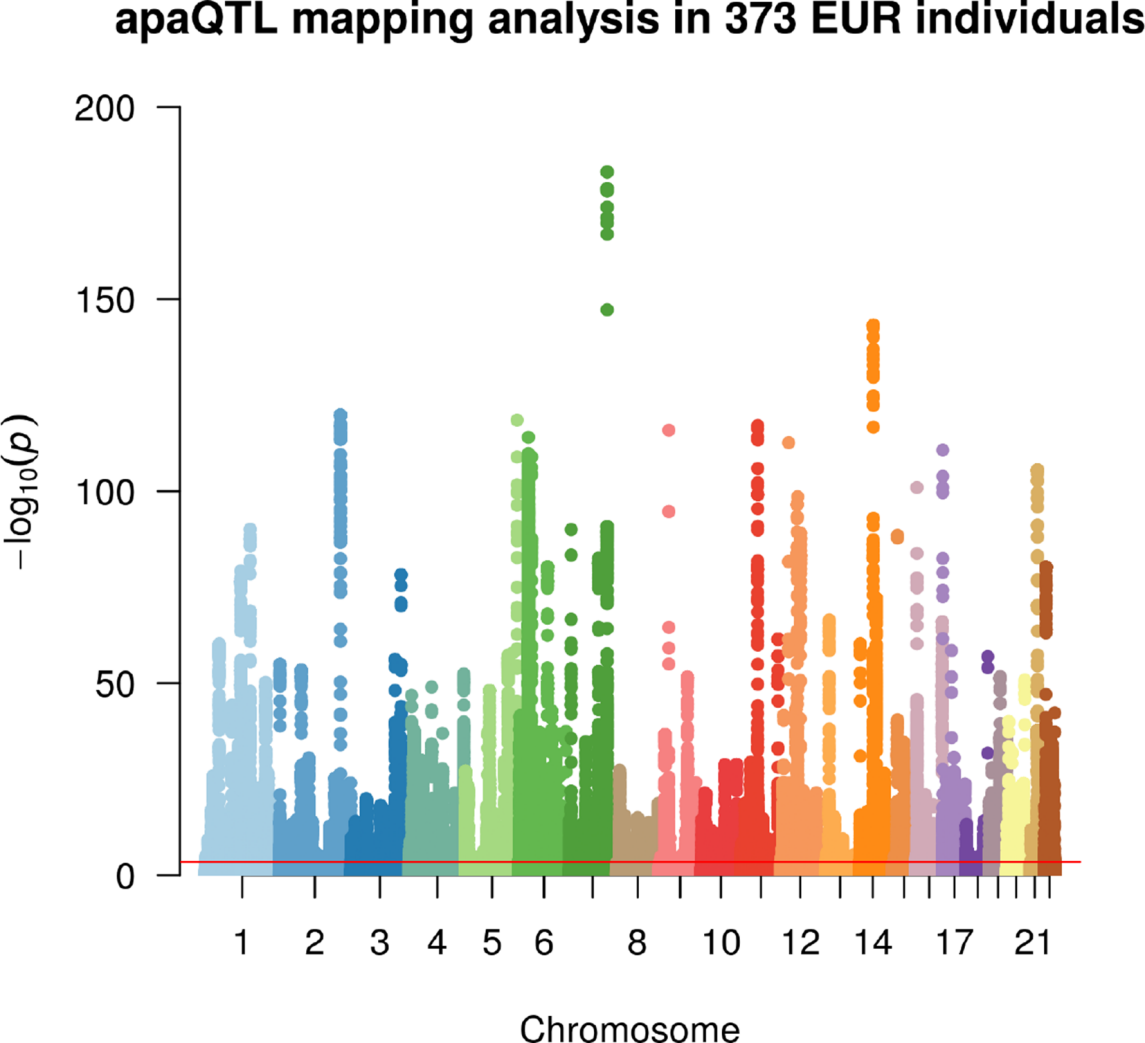
Manhattan plot illustrating the results of the apaQTL mapping analysis. For each fitted model, the −log10 nominal *P* value is shown according to the position of the tested genetic variant. The red line indicates the threshold for genome-wide statistical significance, after multiple-testing correction (nominal *P* < 3.1 × 10^−4^, corresponding to corrected empirical *P* < 0.05).

Linear regression was then used to identify associations between the *m*/*M* values of each gene and the genetic variants within a cis-window including the gene itself and all sequence located within 1 Mbp from the transcription start site (TSS) or the transcription end site (TES). This led to the fitting of ∼30 million linear models, involving ∼6,300 genes and ∼5.3 million variants. About 190,000 models, involving 2,530 genes and ∼160,000 variants, revealed a significant association (**Figure 2**, **Table 1**, **File S1** and **File S2**).

**Table 1:** Results of alternative polyadenylation quantitative trait loci (apaQTL) mapping analysis.

	Total	Significant
Models	30,136,480	192,715
Genes	6,256	2,530
Variants	5,309,860	160,223

**Figure 3:**
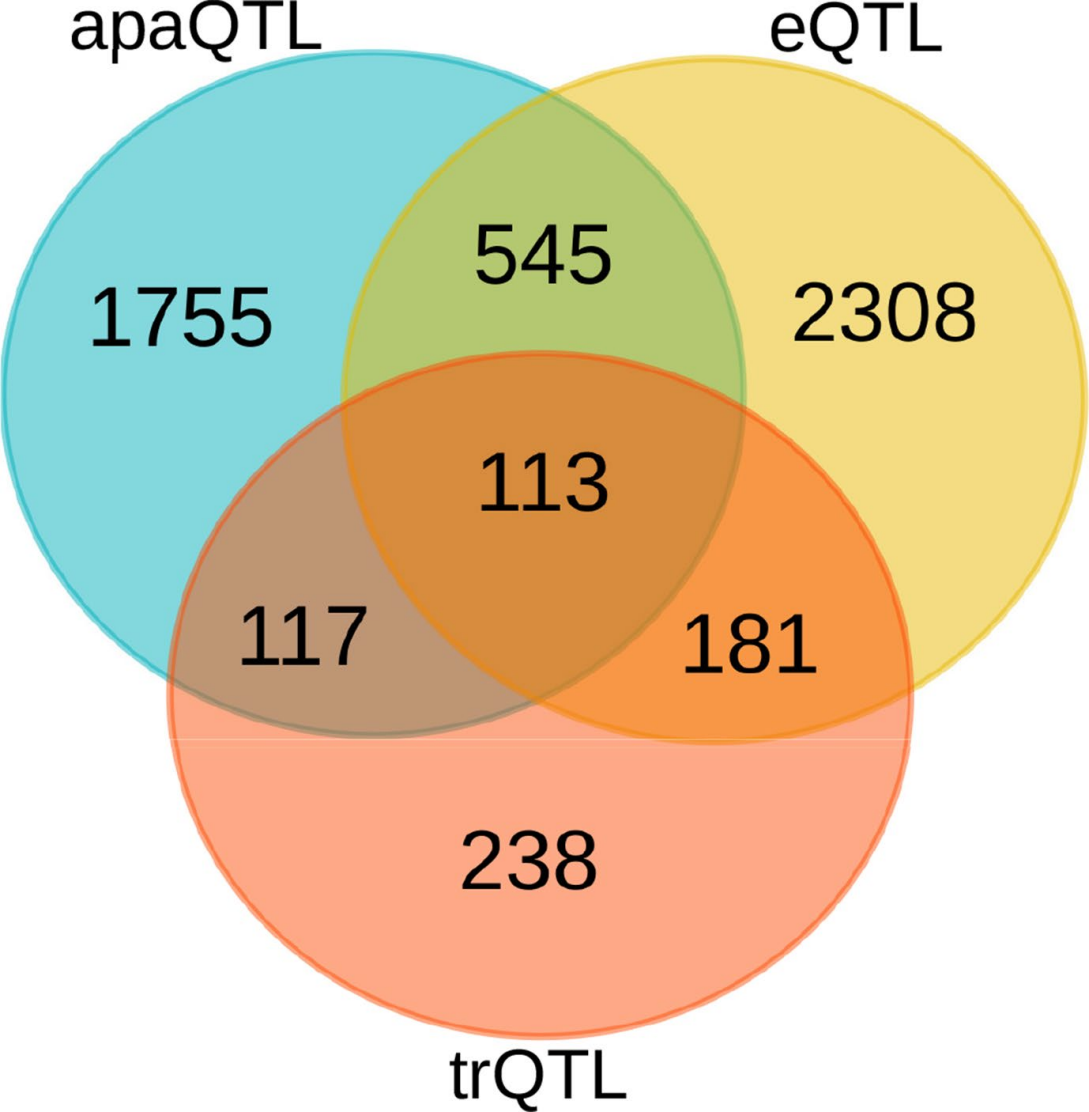
Comparison of genes with different molecular QTLs. Overlap between genes with significant alternative polyadenylation QTL (apaQTL), expression QTL (eQTL), and transcript ratio QTL (trQTL).

Our set of significant genes shows only moderate overlap with genes, for which eQTLs or transcript ratio QTLs (trQTLs) were reported in Lappalainen et al. (2013) from the same data (**Figure 3**). Alternative polyadenylation can result in changes in gene expression levels as a consequence of the isoform-dependent availability of regulatory elements affecting the stability of transcripts, such as microRNA binding sites (Tian and Manley, 2017). In this case, apaQTLs should also be eQTLs. However, APA may also have effects that do not imply changes in expression levels, including the modulation of mRNA translation rates (Spies et al., 2013; Floor and Doudna, 2016) and localization (An et al., 2008), and protein cytoplasmic localization (Berkovits and Mayr, 2015). Similarly, a complete overlap with trQTLs is not expected because they were identified by taking into account all the annotated alternative transcripts of a gene including alternative splicing and transcription initiation. The identification of apaQTLs for several genes for which trQTLs were not identified suggests that focusing on a specific class of transcript structure allows higher sensitivity.

These results show that a large number of genetic determinants of alternative polyadenylation can be inferred from the analysis of standard RNA-Seq data paired with the genotypic characterization on the same individuals.

### apaQTLs Are Preferentially Located Within Active Genomic Regions

**Figure 4:**
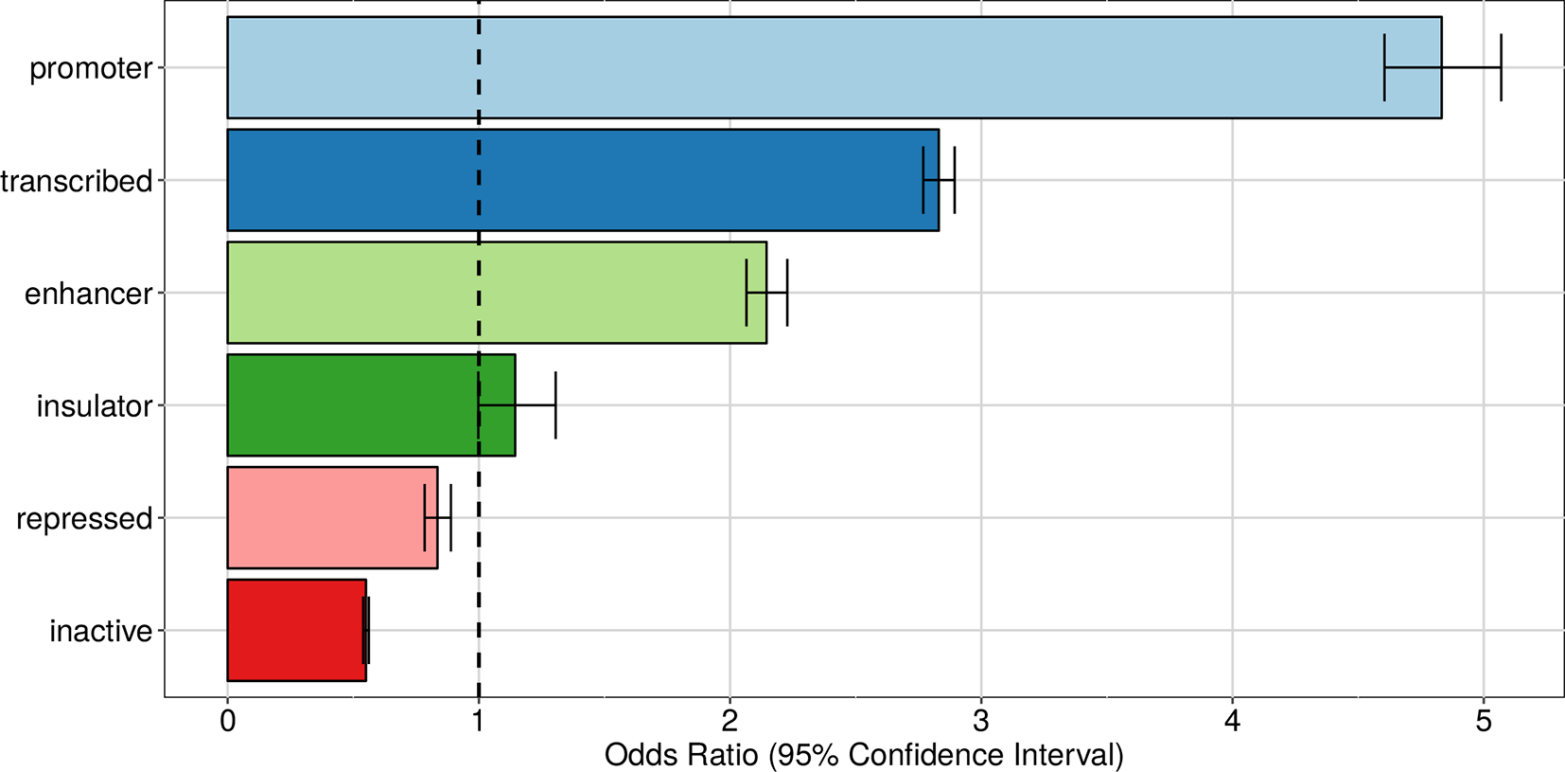
Enrichment of apaQTLs within active genomic regions in the GM12878 cell line. For each broad state, that was defined starting from the ChromHMM annotation, the odds ratio (OR) obtained by logistic regression and its 95% CI are shown.

Just like eQTLs, we expect apaQTLs be located within genomic regions that are active in the relevant cell type (lymphoblastoid cells for our data). To verify this hypothesis, we superimposed the apaQTLs to the ChromHMM annotation of the human genome for the GM12878 cell line (Ernst et al., 2011) and used logistic regression, as detailed in the *Material and Methods*, to determine the enrichment or depletion of apaQTLs for each chromatin state, expressed as an odds ratio (OR). As expected, significant ORs >1 were obtained for active genomic regions, such as transcribed regions, promoters, and enhancers, suggesting that genetic variants have a higher probability of being apaQTLs when they are located in active regions. Conversely, apaQTLs were depleted in repressed and inactive chromatin states. Similar results were obtained using broad chromatin states (**Figure 4**), defined following Ernst et al. (2011) or all 15 chromatin states reported by ChromHMM (**Figure S1**).

As a control, the same enrichment analysis was performed with the ChromHMM annotation obtained in a different cell type, namely, normal human epithelial keratinocytes (NHEK). All NHEK active chromatin states showed a reduced enrichment in apaQTLs compared with GM1278, and regions repressed in NHEK cells actually showed significant enrichment of lymphoblastoid apaQTLs (**Figure S2** and **Figure S3**). Taken together, these results show that genetic variants affecting alternative polyadenylation tend to be located in cell-type-specific active chromatin regions.

The detection of a significant apaQTL enrichment within promoters and enhancers suggests that also these genomic regions may be involved in the APA regulation, in agreement with the similar enrichment found, generically for trQTLs, in Lappalainen et al. (2013). However, these results could also be explained, in principle, by linkage disequilibrium between promoters or enhancers and 3′ UTR regions. To evaluate the prevalence of this phenomenon, we observed that among 2,113 (3,192) significant genetic variants surviving linkage disequilibrium (LD) pruning (see *Material and Methods*) inside promoters (enhancers), only 288 (376) are in LD (*R* ^2^ > 0.8) with significant genetic variants within 3′ UTRs. Furthermore, the reported enrichments remained highly significant after the exclusion of these variants, supporting the idea that promoters and enhancers have an independent role in the genetic component of APA regulation.

In the following, we will divide apaQTLs in two classes: intragenic apaQTLs are those located inside one of the genes whose isoform ratio we are able to analyze, while all other apaQTLs will be referred to as extragenic (note that these might be located inside a gene for which we are unable to perform the analysis, for one of the reasons explained in the *Material and Methods*).

### Intragenic apaQTLs Are Enriched in Coding Exons and 3′ UTRs

**Figure 5:**
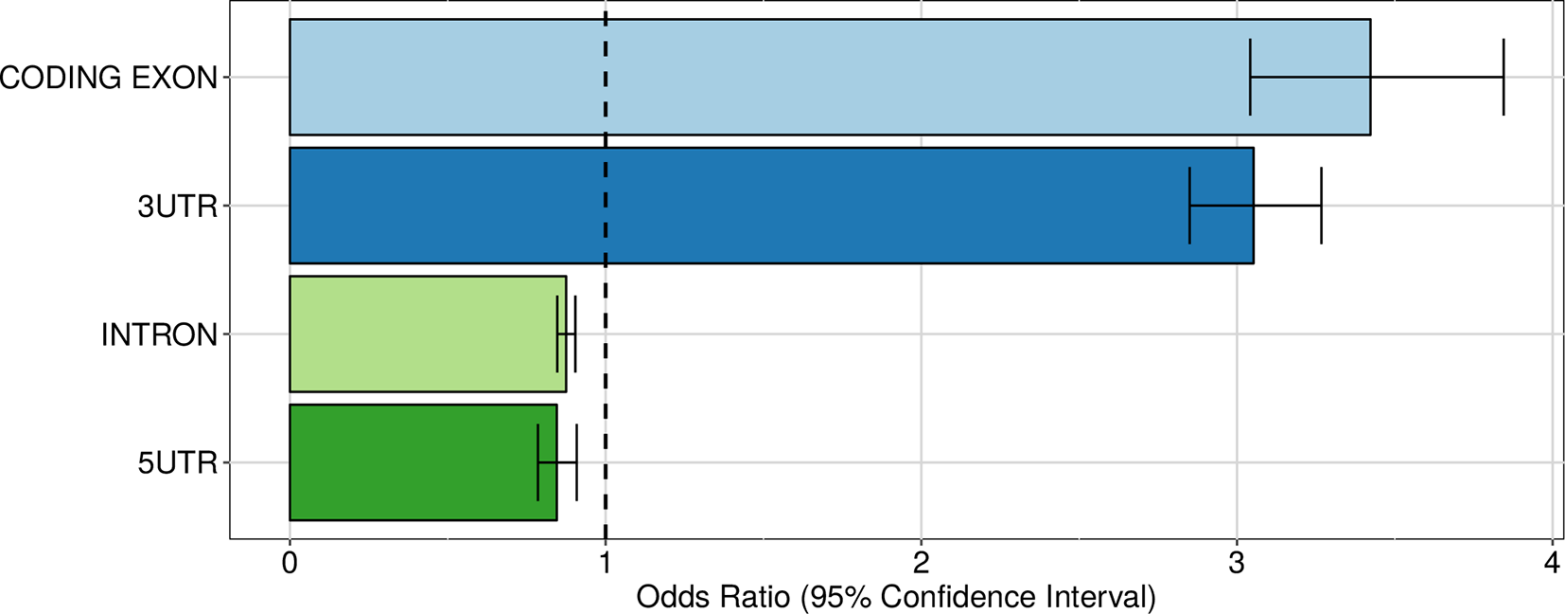
Enrichment of intragenic apaQTLs within coding and noncoding transcript regions. For each gene region, the OR obtained by logistic regression and its 95% CI are shown.

Having established that genetic variants have a widespread influence of the expression of alternative 3′ UTR isoforms, we turned to their putative mechanisms of action. First of all, we considered the distribution of intragenic apaQTLs among regions contributing to the mRNA versus introns. As shown in **Figure 5**, intragenic apaQTLs are enriched in coding exons and 3′ UTRs and depleted in introns and 5′ UTRs. The depletion of introns suggests that most intragenic apaQTLs exert their regulatory role at the transcript level, e.g., by modulating the binding of trans-acting factors to the mRNA.

Among mRNA regions, the enrichment of 3′ UTRs is expected, since these regions contain several elements involved in the regulation of both alternative polyadenylation and mRNA stability. The enrichment of coding exons could be ascribed to regulatory elements residing in these portions of the mRNAs or to residual effects of LD with variants located in the 3′ UTR, notwithstanding the LD pruning procedure implemented in the enrichment analysis (see *Material and Methods*). Note that while several poly(A) sites are located upstream of the last exon (Tian et al., 2007), within both intronic sequences and internal exons, such sites were not taken into account in our analysis. Finally, the depletion of 5′ UTRs might be due to the distance of these elements from the polyadenylation loci and to the fact that these regions are mostly involved in other regulatory mechanisms, such as translational regulation (Hinnebusch et al., 2016). In the following, we examine in more detail three possible mechanisms by which intragenic apaQTLs could exert their action.

#### Creation and Destruction of PAS Motifs

The first possibility is direct interference with the APA regulation, favoring the production of one of the two isoforms in individuals with a particular genotype. A comprehensive atlas of high-confidence PAS has been recently reported (Gruber et al., 2016). In addition to the canonical PAS motifs (AAUAAA and AUUAAA), it contains 10 previously known signals and 6 new motifs. Exploiting this resource, we were able to identify SNPs that cause the creation or the destruction of putative functional PAS motifs, and, as expected, we found that they were enriched among apaQTLs [OR = 1.72, 95% confidence interval (CI) = 1.08−2.75, *P* = 0.0216]. In total, 42 PAS-altering variants were found to be apaQTLs of the gene in which they reside. While expected, this result can be considered to validate our strategy.

**Figure 6:**
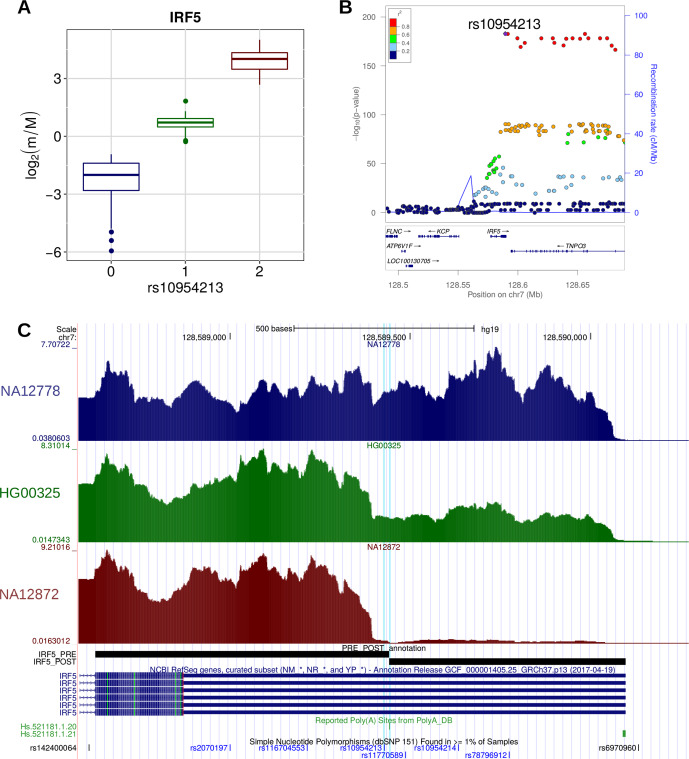
**(A)** Boxplot showing the variation of the log2-transformed *m*/*M* values obtained for *IRF5* as a function of the genotype of the individuals for rs10954213. **(B)** LocusZoom plot (Pruim et al., 2010) illustrating the results obtained for *IRF5* in the genomic region around rs10954213 (100 kb both upstream and downstream its genomic location). In the top panel, each tested genetic variant was reported as a function of both its genomic coordinate and its association level with *IRF5* (log10-transformed nominal *P* value); the points color reflects the linkage disequilibrium (LD) level (*R* ^2^) between rs10954213 and each of the other genetic variants in the locus. The bottom panel shows the genes and their orientation in the locus. **(C)** Figure adapted from the UCSC Genome Browser screenshot. RNA-Seq tracks, reporting coverage per million mapped reads, are shown for three representative individuals: NA12778 (homozygous for the reference allele), HG00325 (heterozygous) and NA12872 (homozygous for the alternative allele). *IRF5* RefSeq, *IRF5* PRE/POST segments, poly(A) sites, and common SNPs are shown. The rs10954213 variant and the affected poly(A) site (Hs.521181.1.20) are highlighted.

A few examples are worth discussing in detail. SNP rs10954213 was shown by several studies (Cunninghame Graham et al., 2007; Graham et al., 2007; Yoon et al., 2012) to determine the preferential production of the short isoform of the *IRF5* transcription factor through the conversion of an alternative PAS motif (AAUGAA) into the canonical one (AAUAAA) in a proximal position within the 3′ UTR. Consistently, we found that this variant is associated with higher prevalence of the short isoform (**Figure 6**). Moreover, the same variant was associated to higher risk of systemic lupus erythematosus (SLE) and higher *IRF5* expression, which could be due to the loss of AU-rich elements (ARE) in the short transcript isoform (Yoon et al., 2012). Globally, these findings are in agreement with the known involvement of *IRF5* in several pathways that are critical for the onset of SLE [type I IFN production, M1 macrophage polarization, autoantibody production, and induction of apoptosis (Lazzari and Jefferies, 2014)].

**Figure 7:**
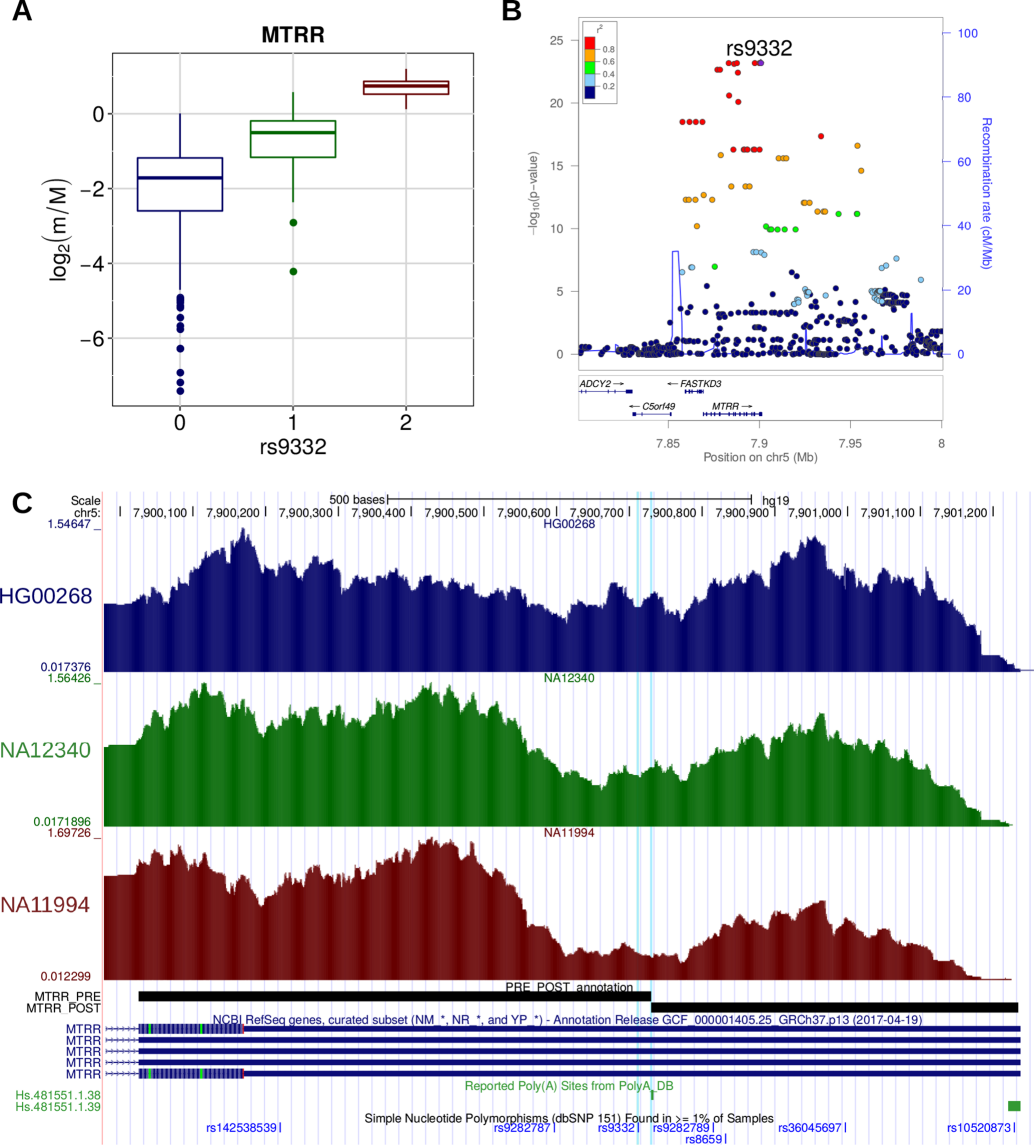
**(A)** Boxplot showing the variation of the log2-transformed *m*/*M* values obtained for *MTRR* as a function of the genotype of the individuals for rs9332. **(B)** LocusZoom plot illustrating the results obtained for *MTRR* in the genomic region around rs9332 (100 kb both upstream and downstream its genomic location). **(C)** Figure adapted from the UCSC Genome Browser screenshot. RNA-Seq tracks, reporting coverage per million mapped reads, are shown for three representative individuals: HG00268 (homozygous for the reference allele), NA12340 (heterozygous), and NA11994 (homozygous for the alternative allele). *MTRR* RefSeq, *MTRR* PRE/POST segments, poly(A) sites, and common SNPs are shown. The rs9332 variant and the affected poly(A) site (Hs.481551.1.38) are highlighted.

A similar trend was detected in the case of the rs9332 variant, located within the 3′ UTR of the *MTRR* gene, encoding an enzyme essential for methionine synthesis (**Figure 7**). This variant was reported to be associated with a higher risk of spina bifida, along with other variants within the same gene (Shaw et al., 2009). We found that the variant is associated with the increased relative expression of the short isoform of the *MTRR* transcript, as a consequence of the creation of a proximal canonical PAS. We can thus speculate that, similarly to what was shown for *IRF5*, this posttranscriptional event could lead to a variation in the activity of the enzyme activity and ultimately to increased disease susceptibility.

The same mechanism might provide putative mechanistic explanations for associations found by GWAS studies. For example, we found the variant rs5855 to be an apaQTL for the *PAM* gene (**Figure S4**), essential in the biosynthesis of peptide hormones and neurotransmitters (Eipper et al., 1983; Czyzyk et al., 2005; Gaier et al., 2014). No eQTLs or trQTLs for this gene were revealed by the analysis of the same data reported in Lappalainen et al. (2013). This variant replaces an alternative PAS motif (AGUAAA) with the canonical AAUAAA, thus presumably increasing its strength. This PAS motif is located 26 bps upstream of an APA site corresponding to a 3′ UTR of ∼450 bps, instead of the ∼2,000 bps of the canonical isoform, lacking several predicted microRNA binding sites. Indeed, our analysis revealed a shortening of the 3′ UTR in individuals with the alternate allele, i.e., the canonical PAS motif. Notably, the variant is in strong LD (*R* ^2^ = 0.90) with the intronic variant rs10463554, itself an apaQTL for *PAM*, which has been associated to Parkinson’s disease in a recent meta-analysis of GWAS studies (Chang et al., 2017a).

Conversely, the destruction of a canonical, proximal PAS motif leads to shortening of the 3′ UTR of *BLOC1S2* (**Figure S4**). The variant rs41290536 replaces the canonical PAS motif AAUAAA with the noncanonical one AAUGAA 17 bps upstream of a poly(A) site corresponding to a UTR length of ∼750 bps compared to the ∼2,200 of the longest isoform. The variant is in complete LD (*R* ^2^ = 1) with two variants that have been associated to predisposition to squamous cell lung carcinoma (rs28372851 and rs12765052) (McKay et al., 2017).

#### Alteration of MicroRNA Binding

In an alternative scenario, genetic variants can influence the relative expression of alternative 3′ UTR isoforms by acting on the stability of transcripts, for example through the creation or destruction of microRNA binding sites. For each gene with alternative 3′ UTR isoforms, we divided the 3′ UTR into two segments: the “PRE” segment, common to both isoforms, and the “POST” segment, contained only in the longer isoform. Variants altering microRNA binding sites located in the POST segment can result in the variation of the relative isoform expression since they affect only the expression of the long isoform.

For example, we found that the rs8984 variant is associated with an increased prevalence of the long transcript isoform of the *CHURC1* gene, an effect that could be due to the destruction of a binding site recognized by microRNAs of the miR-582-5p family within the POST segment of the gene (**Figure S5**). More generally, we found that apaQTLs are enriched, albeit slightly, among the genetic variants that create or break putative functional microRNA binding sites (OR = 1.15, 95% CI = 1.02−1.30, *P* = 0.022). However, we could not find significant agreement between the predicted and actual direction of the change in isoform ratios for these cases. Together with the marginal significance of the enrichment, this result suggests that the alteration of microRNA binding sites is not among the most relevant mechanisms in the genetic determination of 3′ UTR isoform ratios.

#### Alteration of RNA-Protein Binding

RNA-binding proteins (RBPs) play important roles in the regulation of the whole cascade of RNA processing, including co- and posttranscriptional events. Although many of them have not been fully characterized yet, a collection of 193 positional weight matrices (PWMs) describing a large number of RNA motifs recognized by human RBPs has been obtained through *in vitro* experiments (Ray et al., 2013). Here, we exploited this resource to identify SNPs that alter putative functional RBP binding sites. Consistently with the involvement of RBPs in the regulation of alternative polyadenylation, mRNA stability, and microRNA action, we found a highly significant enrichment of RBP-altering SNPs among intragenic apaQTLs (OR = 1.48, 95% CI = 1.31–1.66, *P* = 8.54 × 10^−11^).

Specifically, we obtained a positive and significant OR for 20 individual RBP-binding motifs (**Table S1**). Although in most cases the enrichment is modest, some of the enriched motifs correspond to RNA-binding domains found in RBPs with a previously reported role in polyadenylation regulation [members of the muscle blind protein family (Shi and Manley, 2015; Ha et al., 2018), *KHDRBS1* (La Rosa et al., 2016), and *HNRNPC* (Gruber et al., 2016)]. Other enriched RNA-binding motifs are associated with splicing factors (*RBM5*, *SRSF2*, *SRSF9*, and *RBMX*) and other RBPs that may be involved in RNA processing (such as members of the MEX3 protein family and HNRNPL). On the contrary, only one significant motif is associated with an RBP that may be involved in RNA degradation [*CNOT4* (Miller and Reese, 2012)]. The involvement of several splicing factors is consistent with evidence supporting a mechanistic interplay between polyadenylation and splicing, which goes beyond the regulation of the usage of intronic poly(A) sites (Gunderson et al., 1994; Lutz et al., 1996; Liang and Lutz, 2006; Millevoi et al., 2006).

### Extragenic apaQTLs Act in-Cis Through the Perturbation of Regulatory Elements

Understanding the function of extragenic apaQTLs is less straightforward because, although there are few examples of DNA regulatory elements contributing to APA regulation (Oktaba et al., 2015), it is commonly believed that APA is mainly controlled by cis-elements located within transcripts, both upstream and downstream of the poly(A) sites (Tian and Manley, 2017).

To further explore this aspect we took advantage of a different annotation of active genome regions, which includes the association between regulatory regions and target genes, namely, the cis-regulatory domains (CRDs) identified in lymphoblastoid cell lines in Delaneau et al. (2019). Extragenic apaQTLs were indeed found to be enriched in CRDs (OR = 1.73, 95% CI = 1.69–1.78, *P* < 10^−16^). The 3D structure of the genome is a key aspect of gene regulation (Krijger and de Laat, 2016), as it determines physical contacts between distal regulatory regions and proximal promoters. In particular, CRDs have been described as active sub-domains within topologically associating domains (TADs), containing several noncoding regulatory elements, both proximal and distal. The perturbation of those regulatory elements by genetic variants can lead to the alteration of gene expression and perhaps interfere with other processes such as alternative polyadenylation, as suggested by our results. Importantly, CRDs have been assigned to the nearby genes they regulate. We could thus observe that extragenic apaQTLs tend to fall within CRDs that have been associated with their target genes much more frequently than expected by chance. Indeed, this correspondence was verified for 27,527 extragenic apaQTLs, while the same degree of concordance was never obtained in 100 permutations in which each extragenic apaQTL was randomly associated to a gene in its cis-regulatory window (median number of correspondences, 12,571). These results suggest an important role of genetic variants located in active, nontranscribed cis-regulatory regions in regulating alternative polyadenylation of the target genes.

### A Role for apaQTLs in Complex Diseases

Since common genetic variation is involved in complex diseases, often by affecting gene regulation, a natural question is whether apaQTLs can be used to provide a mechanistic explanation for some of the genetically driven variability of complex traits, thus adding 3′ UTR length to the list of useful intermediate phenotypes. Besides the specific examples discussed above, we found an overall striking enrichment among apaQTLs of genetic variants reported in the NHGRI-EBI GWAS Catalog (MacArthur et al., 2017) (OR = 3.17, 95% CI = 3.01−3.33, *P* < 10−16).

**Figure 8:**
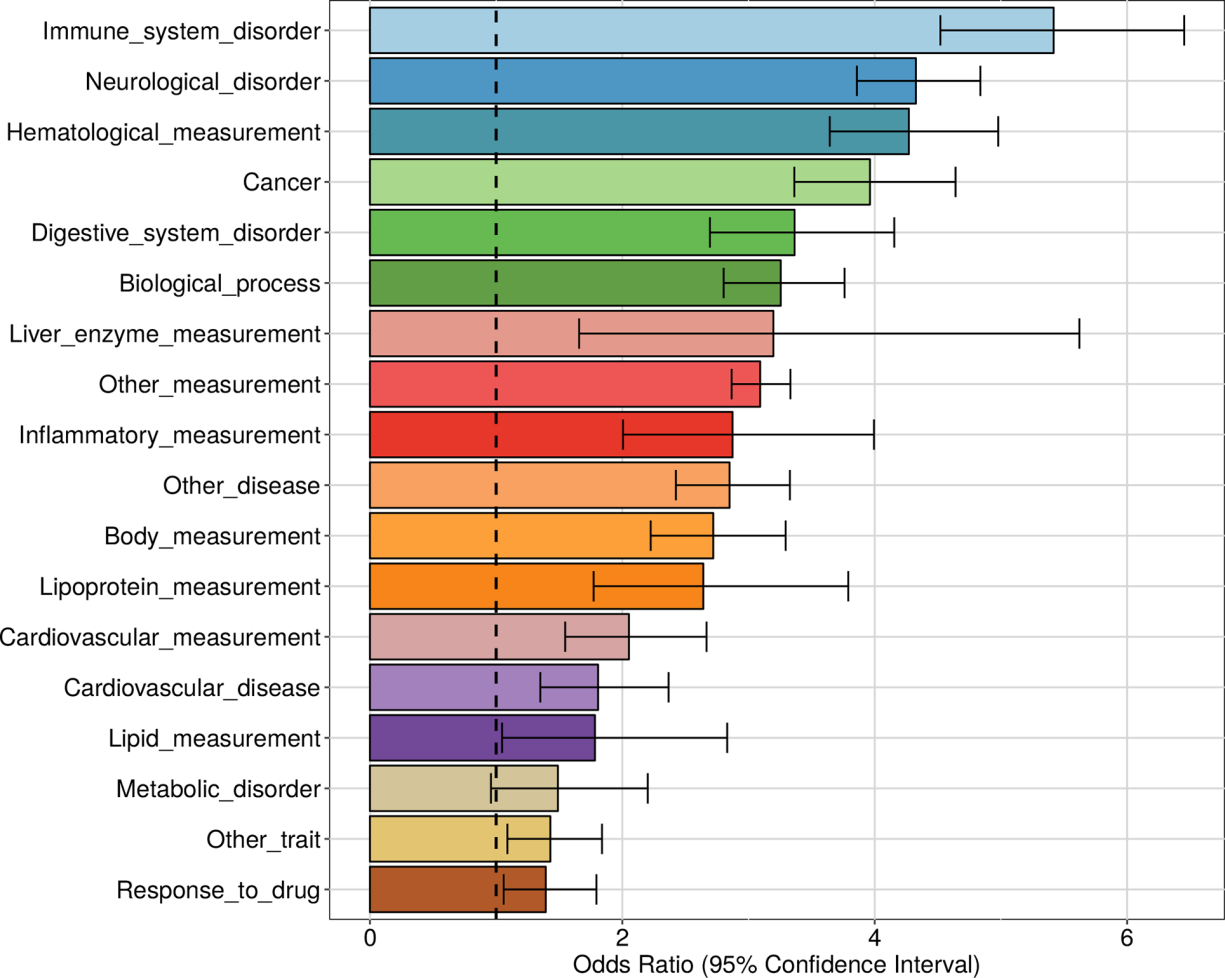
Enrichment of genome-wide association studies (GWAS) hits among apaQTLs, for different categories of complex traits. For each category, the OR obtained by logistic regression and its 95% CI are shown.

We also investigated the enrichment of each trait category defined by the Experimental Factor Ontology (EFO) and then for each individual trait. In line with the fact that the apaQTL mapping was performed in lymphoblastoid cells, the strongest enrichment was observed for immune system disorders (OR = 5.41, 95% CI = 4.52−6.45, *P* = 2.50 × 10^−77^) (**Figure 8** and **Table S2**). However, a strong enrichment was also detected for almost all the other tested categories, including neurological disorders (OR = 4.32, 95% CI = 3.86−4.83, *P* = 2.47 × 10^−142^) and cancer (OR = 3.96, 95% CI = 3.36−4.64, *P* = 4.15 × 10^−63^).

A significant enrichment was detected for 95 individual complex traits, including several diseases. Among these, the largest ORs were observed for autism spectrum disorder (OR = 42.6, 95% CI = 32.9−55.5, *P* = 2.36 × 10^−174^), squamous cell lung carcinoma (OR = 26.1, 95% CI = 15.7−43.3, *P* = 1.29 × 10^−36^), lung carcinoma (OR = 17.9, 95% CI = 12.7−25.2, *P* = 9.63 × 10^−62^), schizophrenia (OR = 10.6, 95% CI = 9.01−12.4, *P* = 1.25 × 10^−182^), and HIV-1 infection (OR = 6.51, 95% CI = 3.75−10.8, *P*= 2.28 × 10^−12^). The complete list of enriched traits can be found in **File S3**.

We observed that apaQTLs that are also GWAS hits often map to genes in the human leukocyte antigen (HLA) locus, suggesting that, in at least some cases, the enrichment could be mostly driven by this genomic region. Somewhat unexpectedly, this was particularly evident for neurological disorders. To clarify this point, we evaluated all enrichments after excluding the variants in the HLA locus. Although in some cases the OR decreased after removing HLA variants, for most GWAS categories, the enrichment was still significant (**Figure S6** and **Table S3**). For example, we found 155 apaQTLs associated with autism spectrum disorder, 116 of which affecting HLA genes. After the exclusion of HLA variants, the enrichment was still highly significant (OR = 10.66, 95% CI = 6.92−15.95, *P* = 7.05 × 10^−29^). On the contrary, the enrichment of variants associated to pulmonary adenocarcinoma is driven by the HLA locus and becomes nonsignificant after excluding HLA variants (OR = 1.35, 95% CI = 0.22−4.39, *P* = 0.68). The complete list of enriched traits after the exclusion of HLA variants can be found in **File S4**.

### The Effect of Genetic Variants on APA Can be Confirmed in Patients

As briefly discussed above, the rs10954213 variant is associated with a higher risk of SLE. Evidence about the related molecular mechanism arose from the analysis of cell lines derived from healthy individuals (Cunninghame Graham et al., 2007; Graham et al., 2007), and the effect of the variant on *IRF5* expression in blood cells was confirmed in SLE patients (Kozyrev et al., 2007; Feng et al., 2010). However, direct evidence on the effect of this variant on APA regulation in SLE patients is still missing.

**Figure 9:**
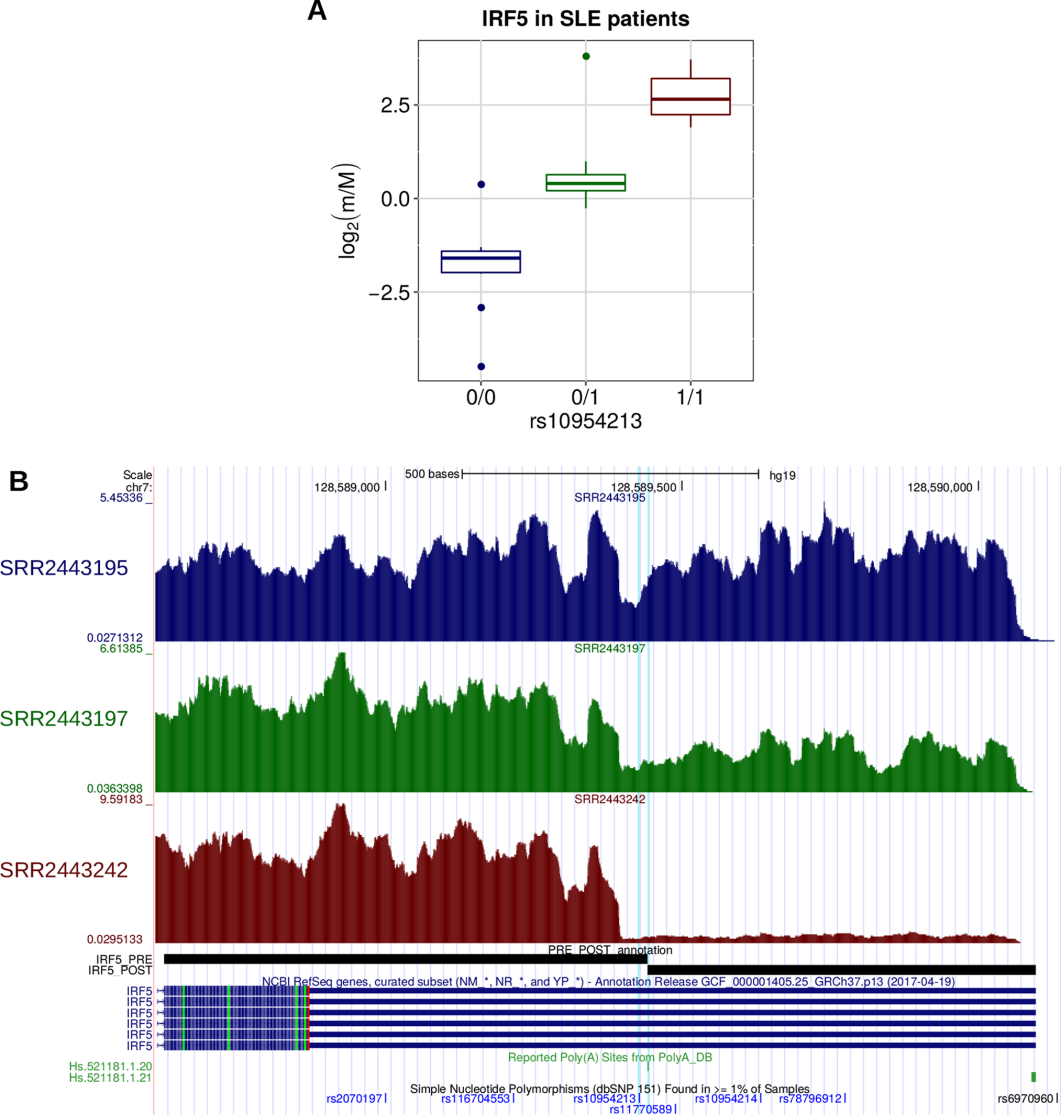
**(A)** The effect of rs10954213 on the relative expression of the *IRF5* alternative isoforms was investigated also in a small cohort of systemic lupus erythematosus (SLE) patients. The boxplot shows the variation of the log2-transformed *m*/*M* values obtained for *IRF5* as a function of the genotype of the individuals. **(B)** Figure adapted from the UCSC Genome Browser screenshot. RNA-Seq tracks, reporting coverage per thousand mapped reads, are shown for three representative individuals: SRR2443195 (homozygous for the reference allele), SRR2443197 (heterozygous), and SRR2443242 (homozygous for the alternative allele). *IRF5* RefSeq, *IRF5* PRE/POST segments, poly(A) sites, and common SNPs are shown. The rs10954213 variant and the affected poly(A) site (Hs.521181.1.20) are highlighted.

To assess whether rs10954213 affects *IRF5* APA regulation in SLE patients, we analyzed RNA-Seq data derived from whole blood cells in 99 patients (Hung et al., 2015). After the exclusion of 52 individuals whose genotype cannot be determined with certainty from RNA-Seq reads, we detected a strong difference in *IRF5* *m*/*M* values among the three rs10954213 genotypes, with the alternative allele associated with higher *m*/*M* values, i.e., shorter 3′ UTR (Kruskal−Wallis test *P* = 2.49 × 10^−8^; **Figure 9**). Therefore, the variant has, at least qualitatively, the same effect in the whole blood of SLE patients as in lymphoblastoid cell lines of normal individuals.

## Discussion

We used a new efficient strategy to study how human genetic variants influence the expression of alternative 3′ UTR isoforms. This issue has been previously investigated with different approaches (Kwan et al., 2008; Thomas and Sætrom, 2012; Lappalainen et al., 2013; Zhernakova et al., 2013; Monlong et al., 2014). The method we propose combines wide applicability, being based on standard RNA-Seq data, with the high sensitivity allowed by limiting the analysis to a single type of transcript structure variant, namely, 3′ UTR length. Such higher sensitivity led us to discover thousands of variants associated with 3′ UTR length that were not identified in a general analysis of transcript structure from the same data in Lappalainen et al. (2013). Moreover, the significant overlap between our apaQTLs and the eQTLs identified in Lappalainen et al. (2013) confirms the known relevant role of 3′ UTRs in gene expression regulation. However, the regulation of 3′ UTR length is known to affect regulatory processes that do not directly alter mRNA abundance, such as regulation of translation efficiency, mRNA localization, and membrane protein localization (Elkon et al., 2013; Berkovits and Mayr, 2015). Indeed, most of the apaQTLs we found were not identified as eQTLs in Lappalainen et al. (2013).

The various mechanisms underlying the association between genetic variants and the relative abundance of 3′ UTR isoforms can be classified in two main classes based on whether they affect the production or degradation rates of the isoforms. The production-related mechanisms include the alteration of APA sites, of cis-regulatory elements located in promoters and enhancers, and of binding sites of RBPs involved in nuclear RNA processing; the degradation-related mechanisms include the alteration of the binding sites of microRNAs and cytoplasmatic RBPs affecting mRNA stability. Taken together, our results suggest that the genetic effects on 3′ UTR isoforms act prevalently at the level of production, as shown by the strong enrichment of apaQTLs in nontranscribed regulatory regions and among the variants creating or disrupting APA sites and by the relatively weak enrichment of variants creating or disrupting microRNA binding sites. In addition, the results on altered RBP binding sites confirm this picture, since most motifs altered by apaQTLs are associated to nuclear RBPs involved in nuclear RNA processing.

In particular, we identified several apaQTLs creating or destroying putative functional PAS motifs. However, it should be noted that our ability to detect these events is intrinsically limited by the motif repertoire that we used (Gruber et al., 2016), which might miss some of the rarest alternative PAS motifs. For example, we found that the rs6151429 variant is associated with the increased expression of the long isoform of the transcript codified by the Arylsulfatase A (*ARSA*) gene (**Figure S4**), in agreement with previous evidence (Gieselmann et al., 1989). However, we did not include this variant among those disrupting a PAS motif since the disrupted motif (AAUAAC) is not included in the catalog that we used. In addition, we considered only PAS-altering single nucleotide substitutions, while also other types of genetic variants can modify the PAS landscape of a gene. For example, a small deletion (rs374039502) causes the appearance of a new PAS motif within the *TNFSF13B* gene and has been associated with a higher risk of both multiple sclerosis and SLE in the Sardinian population (Steri et al., 2017).

We observed a strong enrichment of apaQTLs in regulatory regions such as promoters and enhancers, as previously found for variants generically affecting transcript structure in Lappalainen et al. (2013). These results point to an important role of DNA-binding cis-acting factors in the regulation of 3′ UTR length and to the existence of a widespread coupling between transcription and polyadenylation (Ji et al., 2011; Elkon et al., 2013). The mechanisms behind this coupling are thought to include the interaction between rates of Pol II elongation and alternative polyadenylation and the recruitment, by the transcription machinery, of trans-acting factors affecting PAS choice (Tian and Manley, 2017). Moreover, it has been shown that RBPs involved in APA regulation can interact with promoters (Oktaba et al., 2015).

Regarding the effect of genetic variants on mRNA stability, we focused on the perturbation of microRNA binding, taking into account both the creation and the destruction of microRNA binding sites within transcripts. The relevance of mRNA stability seemed to be confirmed by a modest enrichment of microRNA-altering SNPs among intragenic apaQTL; however, the direction of their effect on microRNA binding is not statistically consistent with the expected direction of the change in 3′ UTR isoform ratio. The same type of ambiguity has been previously reported with regard to the relationship between the effect of SNPs on microRNA binding and gene expression levels (Võsa et al., 2015) and makes us doubt whether these microRNA-altering apaQTLs are truly causal for the associated gene. These results suggest that the alteration of microRNA binding may not be a predominant mechanism explaining the variation of the expression of alternative 3′ UTR isoforms across individuals. Limitations in the accuracy of predicted micorRNA binding sites might also contribute to this result.

Another possible mechanism of action of intragenic apaQTLs is the perturbation of the regulatory action of RBPs, as indicated by the modest but highly significant enrichment of SNPs altering RNA-binding motifs. However, the lack of strong enrichments when considering each motif individually suggests that specific RBP motifs may have a small regulatory impact on APA that may also depend on the context, as recently suggested (Ha et al., 2018). As in the case of microRNAs, also our limited knowledge of the binding preferences of RBPs might limit our power to detect their effects: More sophisticated models should take into account the highly modular structure of RBPs that often include multiple RNA-binding domains (RBDs), the emerging importance of both the binding context and the RNA structure and even more sophisticated modes of RNA binding (Dominguez et al., 2018; Hentze et al., 2018).

Furthermore, it is reasonable to assume that also noncanonical modes of APA regulation can be affected by genetic variants and therefore drive the detection of variable isoform expression ratios. For example, it has been recently suggested that an epitranscriptomic event, the m6A mRNA methylation, can be associated with alternative polyadenylation (Yue et al., 2018). In addition, recently published results suggest that genetic variants could affect APA regulation also in an indirect way, without affecting the regulatory machinery. Past studies have reported that a narrow range of 10–30nt between the PAS and the poly(A) site is required for efficient processing; however, Wu and Bartel (2017) suggested that also greater distances can sometimes be used, thanks to RNA folding events that bring the PAS and the poly(A) site closer to each other. Therefore, we can speculate that, if a genetic variant affects RNA folding in such a way as to modify the distance between the PAS and the poly(A) site, it could also influence APA regulation.

While the mechanisms discussed above act at the level of the primary or mature transcript, our results revealed a perhaps unexpectedly large number of extragenic apaQTLs, mostly located in regulatory regions. These apaQTLs point to an important role of DNA-binding elements such as transcription factors in regulating alternative polyadenylation through long-distance interactions with cleavage and polyadenylation factors. The investigation of these mechanisms is thus a promising avenue of future research.

Alternative polyadenylation can affect several biological processes, influencing mRNA stability, translation efficiency, and mRNA localization (Tian and Manley, 2017). Therefore, it is not surprising that its perturbation has been associated with multiple pathological conditions (Chang et al., 2017b; Manning and Cooper, 2017). In the present study, we detected a strong enrichment of GWAS hits among apaQTLs, supporting the idea that 3′ UTR length is a useful addition to the list of intermediate molecular phenotypes that can be used for a mechanistic interpretation of GWAS hits. In particular, we identified genetic variants previously associated to neurological disorders, such as autism, schizophrenia, and multiple sclerosis, which may act by affecting the regulation of polyadenylation. The importance of posttranscriptional events in the onset of neurological diseases has been recently confirmed by two studies, demonstrating that genetic variants affecting alternative splicing (sQTL) give a substantial contribution to the pathogenesis of schizophrenia (Takata et al., 2017) and Alzheimer’s disease (Raj et al., 2018). We also observed that the relevant apaQTLs often map to HLA genes but that the enrichment is not explained by the HLA locus alone. On the other hand, examples of APA events involving HLA genes have been reported (Hoarau et al., 2004; Kulkarni et al., 2017), and genes encoding antigen-presenting molecules account for the highest fraction of genetic risk for many neurological diseases (Misra et al., 2018).

A gene-based alternative approach to the interpretation of GWAS has been recently proposed. In the original implementation of Transcriptome Wide Association Studies (TWAS) (Gamazon et al., 2015), eQTL data obtained in a reference dataset are used to predict the genetic component of gene expression in GWAS cases and controls, which is then correlated with the trait of interest, thus allowing the identification of susceptibility genes. More recently, Gusev et al. (2016) proposed a summary-based TWAS strategy in which the association between the genetic component of gene expression and a trait is indirectly estimated through the integration of SNP–expression, SNP–trait, and SNP–SNP correlation data. Furthermore, this kind of analysis has also been performed exploiting a collection of sQTLs, leading to the identification of new susceptibility genes for schizophrenia (Gusev et al., 2018) and Alzheimer’s disease (Raj et al., 2018). In a similar way, apaQTLs could be used to discover cases in which the association between genes and diseases is driven by the alteration of the expression of alternative 3′ UTR isoforms.

We are aware of some limitations of this study. First, the simple model that we used for the definition of alternative 3′ UTRs isoforms limits the type of events that can be detected because we can see only events involving poly(A) sites located within the transcript segments taken into account for the computation of the *m*/*M* values (the PRE and the POST segments). Nonetheless, the adoption of this simple model significantly reduces the computational burden and might be sufficient to indicate general trends that can be subsequently further investigated with more sophisticated models. Indeed, it has been previously shown, in a slightly different context (i.e., the comparison of APA events detected in different cellular conditions or tissues), that the results obtained with our model are comparable with those obtained exploiting a more complex model that takes into account all the possible APA isoforms of a gene, especially because also genes with multiple poly(A) sites mainly use only two or a few of them (Grassi et al., 2016). Second, our strategy depends on a preexisting annotation of poly(A) sites. Methods that infer the location of poly(A) sites from RNA-Seq data are available, but they can have lower sensitivity in the detection of APA events (Grassi et al., 2016; Ha et al., 2018). In addition, although the method is generally able to successfully discriminate APA events from alternative splicing events, it may give rise to spurious associations when intron retention is present within the 3′ UTRs, and therefore, such special cases should be inspected with particular attention. Finally, we examined only a single cell type (lymphoblastoid cells) to demonstrate the feasibility of apaQTL mapping analysis. A broader investigation, exploiting data such as those provided by Genotype–Tissue Expression (GTEx) consortium (Aguet et al., 2017), would be particularly valuable. Indeed, APA regulation seems to be significantly tissue specific and global trends of poly(A) sites selection in specific human tissues have been described: for example transcripts in the nervous system and brain are characterized by preferential usage of distal PAS, whereas in the placenta, ovaries and blood the usage of proximal PAS is preferred (Elkon et al., 2013).

In conclusion, we have identified thousands of common genetic variants associated with alternative polyadenylation in a population of healthy human subjects. Alternative polyadenylation is a promising intermediate molecular phenotype for the mechanistic interpretation of genetic variants associated to phenotypic traits and diseases.

## Material and Methods

### Data Sources

#### Human Genome and Transcriptome

The coordinates of the NCBI Reference Sequences (RefSeqs) in the human genome (hg19) were downloaded from the UCSC Genome Browser (09/04/2015) (O’Leary et al., 2016; Casper et al., 2017). The corresponding transcript-gene map was downloaded from NCBI (version 69) and the Bioconductor R package org.Hs.eg.db v3.4.0 (Carlson, 2016) was used to associate each Entrez Gene Id to its gene symbol. In addition, the reference sequence of the hg19 version of the human genome was downloaded from the ENSEMBL database, and a collection of poly(A) sites was obtained from PolyA_DB2 (10/02/2014) (Lee et al., 2007).

ChromHMM annotations (Ernst et al., 2011) were downloaded from the UCSC Genome Browser for the GM12878 and the NHEK cell lines (http://genome-euro.ucsc.edu/cgi-bin/hgFileUi?db=hg19&g=wgEncodeBroadHmm). In addition, the coordinates of cis regulatory domains (CRDs) and their association with genes were downloaded for lymphoblastoid cells from ftp://jungle.unige.ch/SGX/ (Delaneau et al., 2019).

#### WGS and RNA-Seq Data

We exploited the RNA-Seq data obtained by the GEUVADIS consortium in lymphoblastoid cell lines of 462 individuals belonging to different populations, but we considered only 373 individuals with European ancestry (EUR). BAM files were downloaded from the E-GEUV-1 dataset (Lappalainen et al., 2013) in the EBI ArrayExpress archive (https://www.ebi.ac.uk/arrayexpress/files/E-GEUV-1/). We also downloaded genotypic data for the same individuals and the results of the eQTL/trQTL mapping analyses. The downloaded VCF files include genotypes for 465 individuals: among the 462 of them for which also RNA-Seq data are available, the large majority had been previously subjected to whole-genome sequencing (WGS) by the 1000 Genome Project (Phase 1) (The 1000 Genomes Project Consortium, 2015), but the GEUVADIS consortium additionally obtained genomic data for 41 of them through genotyping with single nucleotide polymorphism (SNP) array followed by genotype imputation (Lappalainen et al., 2013). Furthermore, whole-blood RNA-Seq data for 99 individuals affected by SLE were downloaded from the NCBI SRA database (SRP062966) (Leinonen et al., 2011; Hung et al., 2015).

#### Regulatory Motifs and Related Expression Data

Different collections of regulatory motifs were downloaded. A list of 18 PAS motifs was obtained from Gruber et al. (2016), microRNA seeds were downloaded from TargetScan 7.2 (Agarwal et al., 2015), and positional weight matrices (PWMs) describing the binding specificities of RNA-binding proteins were downloaded from the CISBP-RNA dataset (Ray et al., 2013), including both the experimentally determined motifs and those that were inferred from related proteins. In addition, the list of microRNAs and RBPs expressed in lymphoblastoid cells were obtained from the expression data available in the E-GEUV-2 and E-GEUV-1 datasets on the EBI ArrayExpress archive (https://www.ebi.ac.uk/arrayexpress/files/E-GEUV-2/) (Lappalainen et al., 2013).

#### GWAS Catalog

A collection of genomic loci associated with human complex traits was obtained by downloading the NHGRI-EBI GWAS Catalog, v1.0.2 (MacArthur et al., 2017). This resource is continuously updated: the version we used was downloaded on October 10, 2018, and it was mapped to GRCh38.p12 and dbSNP Build 151. From the same website, we also downloaded a file showing the mapping of all the reported traits to the Experimental Factor Ontology (EFO) terms (Malone et al., 2010), including the parent category of each trait (the version of the downloaded file was r2018-09-30). In addition, the dbSNP Build 151 (Sherry et al., 1999) collection of human genetic variants was downloaded for hg19.

### Annotation of Alternative 3′ UTR Isoforms

We considered the human transcripts included in RefSeq and associated them with the corresponding Entrez Gene Id. Moreover, we collapsed together the structures of all the transcripts assigned to a gene, using the union of all the exons of the various transcripts associated to a gene and defining the 3′ or 5′ UTR using, respectively, the most distal coding end and the most proximal coding start. The annotation of the resulting gene structures can be found in the supplementary data (see **File S5**).

The coordinates of the human poly(A) sites were converted from hg17 to hg19 using liftover (Hinrichs et al., 2006) and then combined with the gene structures defined above to define the alternative 3′ UTR isoforms. For the definition of alternative 3′ UTR isoforms, we adopted a simple model taking into account only two alternative poly(A) sites for each gene because previous evidence suggests that also genes with multiple poly(A) sites mainly use only two of them (Grassi et al., 2016). In particular, for each gene, we selected the most proximal poly(A) site among those falling within exons, preferring those located within the 3′ UTR, and the end of the gene as the distal poly(A) site. In this way, we were able to define two segments of interest for each gene: the PRE segment, extending from the beginning of the last exon to the proximal poly(A) site, and the POST segment, from the proximal poly(A) site to the end of the gene. The PRE fragment is assumed to be contained into both the long and the short isoform, while the POST segment should be contained exclusively into the long isoform. The GTF file used for the computation of *m*/*M* values is available as **File S6**.

The relative prevalence of the short and long isoforms are evaluated, as described below, based on the number of RNA-Seq reads falling into the PRE and POST regions. While the whole region from the transcription start site to the proximal poly(A) site could be taken, in principle, as the PRE region, we chose to limit it to the last exon to minimize the confounding effect of alternative splicing.

### Computation of *m*/*M* Values

Using the Bioconductor R package Roar (Grassi et al., 2016), for each gene with alternative 3′ UTR isoforms, we obtained an *m*/*M* value in each individual. The *m*/*M* value estimates the ratio between the expression of the short and the long isoform of a gene in a particular condition and the *m*/*Ma*,*i* of gene *a* in the *i* *_th_* individual is defined as

(1) $$m/M_{a,i}=\frac{l_{\mathrm{POS}T_{a}}\times \#r_{\mathrm{PR}E_{a,i}}}{l_{\mathrm{PR}E_{a}}\times \#r_{\mathrm{POS}T_{a,i}}}-1$$

where $l_{PRE_{a}}$ and $l_{\mathrm{POS}T_{a}}$ are, respectively, the length of the PRE and POST segment of the gene *a*, and $\#r_{\mathrm{PR}E_{a,i}}$ and $\#r_{\mathrm{POS}T_{a,i}}$ are, respectively, the number of reads mapped on the PRE and the POST segment of the gene *a* in the *i* *_th_* individual.

The *m*/*M* values were computed for 14,542 genes for which we were able to define alternative 3′ UTR isoforms. Infinite and negative values of *m*/*M* (that happen when the POST region does not produce any reads, and when the POST region produces more reads than the PRE region after length normalization, respectively) were considered as missing values. Then, only those on autosomal chromosomes (chr1-22) and with <100 missing *m*/*M* values were selected for the following investigation, leaving us with 6,256 genes.

### Genotypic Data Preprocessing

Starting from the downloaded VCF files, we extracted genotypic data for 373 EUR individuals for whom also RNA-Seq data are available using VCFtools (Danecek et al., 2011). In addition, only common genetic variants with minor allele frequency (MAF) > 5% were considered in all the following analyses. The MAF values were computed taking into account that the reference allele reported in the VCF file may not always be the most frequent one in the EUR population considered by itself, and we conservatively attributed the most frequent homozygous genotype to individuals for which the genotype was missing, thus being sure to exclude all the less frequent variants from the analysis. We are aware that these MAF values may be an underestimate of the real ones, and therefore, in all the enrichment analyses (see below for details), we instead used MAF values obtained ignoring individuals with missing data.

### Principal Component Analysis of Genotypic Data

It is known that special patterns of linkage disequilibrium (LD) can cause artifacts when a principal component analysis (PCA) is used to investigate population structure (Price et al., 2008). We filtered out all the genetic variants falling within 24 long-range LD (LRLD) regions whose coordinates were derived from Price et al. (2008). In addition, following Novembre et al. (2008), we performed an LD pruning of the genetic variants using the –indep-pairwise function from PLINK v1.9 (Chang et al., 2015) to recursively exclude genetic variants with pairwise genotypic *R*2 > 80% within sliding windows of 50 SNPs (with a 5-SNP increment between windows). Also in this case, VCFtools (Danecek et al., 2011) was used to apply all these filters to the VCF files, and finally, EIGENSTRAT v6.1.4 (Price et al., 2006) was used to run the PCA on the remaining genotypic data at the genome-wide level.

### apaQTL Mapping

From a statistical point of view, we adopted the same strategy used in standard eQTL mapping analyses (Lappalainen et al., 2013) to identify genetic variants that influence the expression level of the alternative 3′ UTR isoforms of a gene. For each of the 6,256 examined genes, we defined a cis-window as the region spanning the gene body and 1 Mbp from both its TSS and its TES. Then, for each gene, a linear model was fitted, independently for each genetic variant within its cis-window, using the genotype for the genetic variant as the independent variable and the log2-transformed *m*/*M* value of the gene as the dependent variable:

(2) $$\log_{2}(m/M_{a,i})=\beta_{0}+\beta_{1}\times g_{j,i}+\beta_{2}\times I_{i}+\sum_{n=1}^{3}\alpha_{n}\times gPC_{n,i}+\varepsilon_{a}$$

where *log* *_2_*(*m*/*M* *_a,i_*) is the log2 transformed *m*/*M* value computed for the *a* gene in the *i* *_th_* individual, *g* *_j,i_* is the genotype of the *i* *_th_* individual for the *j* *_th_* genetic variant, *I* *_i_* is the imputation status (0–1) of the *i* *_th_* individual, *gPC* *_n,i_* is the value of the *n* *_th_* principal component (PC) obtained from genotypic data for the *i* *_th_* individual, *β*_0_ is the intercept, *β*_1_, *β*_2_, and α*_n_* are the fitted regression coefficients, and ϵ*_a_* is the error term for the gene *a*.

The fitting of the linear models was performed using the CRAN R package MatrixEQTL (Shabalin, 2012). Genotypes were represented using the standard 0/1/2 codification, referring to the number of alternative alleles present in each individual, and matrices with genotypic information were obtained from VCF files exploiting the Perl API (Vcf.pm) included in the VCFtools suite (Danecek et al., 2011). Following Lappalainen et al. (2013), in all our models, we included both the imputation status of the individuals and the first three PCs obtained from genotypic data as covariates, to correct for possible biases due to population stratification (**Figure S7**) or genotype imputation.

The observed distribution of nominal *P* values was compared with the expected one in quantile–quantile plots (Q–Q plots), revealing the expected inflation due to the LD issue (**Figure S8**). A permutation-based procedure was implemented (Churchill and Doerge, 1994): all the models were fitted again after the random shuffling of the *m*/*M* values of each gene across samples; then, for each gene–variant pair, we counted how many times we obtained a random *P* value less than its nominal *P* value and divided this value by the total number of random tests performed. Finally, to control for multiple testing, the empirical *P* values were corrected with the Benjamini–Hochberg procedure (Benjamini and Hochberg, 1995) and models with a corrected empirical *P* < 0.05 were considered statistically significant. Manhattan plots were drawn using the CRAN R package qqman (Turner, 2018).

### Comparison With Other Molecular QTLs

To compare the genes for which we detected one or more apaQTLs with those for which eQTL/trQTL were reported (Lappalainen et al., 2013), we translated the Ensembl Gene IDs (ENSG) to NCBI Entrez Gene IDs using Ensembl v67 (Zerbino et al., 2018) retrieved using the Bioconductor R package biomaRt v2.30 (Durinck et al., 2005, Durinck et al., 2009). Two hundred twenty-nine ENSGs could not be translated with this procedure and were therefore excluded from this analysis.

### Enrichment Analyses

To functionally characterize the apaQTLs, we analyzed the enrichment of several features among such variants, including their genomic location, their ability to alter known regulatory motifs, and their association with complex diseases. All enrichments were evaluated through multivariate logistic regression to allow correcting for covariates. In this section, we provide an overview of the method but refer to the following subsections for details about each analysis.

For each feature, we first established which genetic variants were potentially associated with the feature (for example, only variants in the 3′ UTR can alter microRNA binding sites). Therefore, each enrichment analysis started with the selection of the “candidate variants” that were subsequently subjected to an LD-based pruning to obtain a subset of independent candidate variants [the same strategy was implemented for example in Li et al. (2013) to evaluate the enrichment of GWAS hits among eQTLs]. LD-based pruning was always performed using PLINK with the same parameters used in the case of the PCA of genotypic data (see above) but applied in each case to the candidate variants only. To each candidate variant surviving pruning, we attributed a binary variable indicating whether it has the feature under investigation. Finally, these variants are classified as apaQTLs (i.e., corrected empirical *P* < 0.05 for at least one gene) and null variants (i.e., nominal *P* > 0.1 in all the fitted models). We excluded the “gray area” variants with nominal *P* < 0.1 but empirical corrected *P* > 0.05 as they are likely to contain many false negatives. Finally, we fitted a multivariate logistic model in which the dependent variable is the apaQTL/null status of the variant, and the independent variables are the feature of interest and covariates. The latter always include the MAF of the variant, since variants with higher MAF are more likely to be found as significant apaQTLs, and possibly other covariates depending on the feature under examination (see below).

The logistic model can thus be written as:

(3) $$t_{j}=\beta_{0}+\beta_{1}\times {\mathrm{Feature}}_{j}+\mathrm{covariates}+\epsilon_{j}$$

(4) $$\mathrm{Pr}{\left(\mathrm{apaQTL}\right)}_{j}=\frac{1}{1+\exp^{-t_{j}}}$$

where *Feature* *_j_* is a binary variable indicating whether the genetic variant *j* has the feature of interest, *β*_0_ is the intercept, *β*_1_ is the regression coefficient for the feature, ϵ*_j_* is the error term, and *Pr*(*apaQTL*)*_j_* is the fitted probability that the genetic variant *j* is an apaQTL. As expected, in our models, the regression coefficient of the MAF was always positive. The regression coefficient of the *Feature* term and its associated *P* value were used to establish if having the feature under investigation influences the probability of being an apaQTL and to compute the corresponding odds ratio (OR).

#### Chromatin States

This analysis was performed independently for two cell types (the GM12878 and NHEK cell lines). In both cases, the candidate variants were virtually all the genetic variants for which the apaQTL models were fitted, but we excluded those not associated with any chromatin state and all the structural variants because their length can prevent them from being univocally associated with a chromatin state.

Each of the 15 chromatin states and 6 broad chromatin classes (promoter, enhancer, insulator, transcribed, repressed, and inactive) defined in Ernst et al. (2011), separately for the two cell lines, was treated as a binary feature to be used as a regressor in Eq. (3), with value 1 assigned to the variants falling within a DNA region associated to the given chromatin state. Only the MAF was included in the covariates.

#### Gene Regions

The candidate variants were all the intragenic variants for which the apaQTL models were fitted. We defined as intragenic all variants falling between the start and the end of the gene, plus 1,000 bps after the end (to take into account possible misannotations of the 3′ UTR).

Independent enrichment analyses were performed for the following sequence classes: coding exons, introns, 5′ UTR, and 3′ UTR. For each class, the binary feature used as a regressor was assigned the value 1 for variants falling within the class and 0 otherwise. Only the MAF was included in the covariates.

#### Cis Regulatory Domains

The candidate variants were all the extragenic variants (i.e., all variants that are not intragenic according to the definition given above) for which an apaQTL model was fitted. The binary feature was given value 1 for variants falling within a CRD and 0 otherwise. Besides the MAF, the distance from the nearest gene was included as a covariate, since variants closer to a gene are more likely to be apaQTLs.

To verify that the apaQTLs tend to be included in the CRDs specifically associated to the gene on which they act, we translated the CRD–gene associations provided in Delaneau et al. (2019) into Entrez Gene IDs, and we counted how many genetic variants fall within a CRD associated to at least one gene for which the variant is an apaQTL. This number was then compared with that obtained in the same way after randomly assigning a target gene to each extragenic variant within the cis-window used for apaQTL analysis (100 independent randomizations were used).

#### Alteration of Putative Functional Motifs

Similar strategies were implemented to investigate the alteration of different types of putative functional motifs by intragenic variants. This analysis was restricted to single nucleotide polymorphisms (SNPs), excluding therefore both indels and structural variants. For all SNPs, we reconstructed the sequence of both the reference (REF) and the alternative (ALT) allele in the 20-bp region around each candidate genetic variant to determine whether the ALT allele creates or destroys a functional motif with respect to the REF allele. The functional motifs analyzed included PAS motifs, microRNA binding sites, and RBP binding sites.

To each candidate variant surviving LD pruning we associated, using PLINK, a list of tagging variants with genotypic *R*2 > 80% and a binary feature value of 1 if the candidate variant itself or any of its tagging variant altered a functional motif. The enrichment of apaQTLs among motif-affecting variants was then evaluated with the logistic model described by Eq. 3. In the following, we describe the details of the logistic model for each class of functional motifs.

**PAS motifs.** The PAS motif is always located upstream of its target poly(A) site. It has been suggested that a narrow range of 10–30 nt is required for efficient processing, but recent work suggests that also larger distances can be functional thanks to RNA folding processes bringing the poly(A) site closer to the PAS (Wu and Bartel, 2017). Assuming that a PAS-altering SNP would affect the usage of its nearest poly(A) site, we associated to each intragenic SNP the nearest downstream poly(A) site, selected those for which such poly(A) site was located within the PRE/POST segments, and retained as candidate variants only those whose distance from the corresponding poly(A) site was between 10 and 100 nt. PAS-altering variants were defined as those for which a particular PAS motif was found in either the REF or the ALT sequence, but not in both (note that the interconversion between PAS motifs is considered as well, assuming that they can have different strength).

**microRNA binding sites.** microRNA binding sites located downstream of a poly(A) site, and hence in the POST segment, can affect the relative abundance of the long and short isoforms by allowing the selective degradation of the former by microRNAs. Therefore, we chose as candidate variants all the SNPs within the POST segment of the genes analyzed. Putative microRNA binding sites were classified, as in Agarwal et al. (2015), in three classes: 8mer, 7mer-m8, and 7mer-A1 (matches classified as 6-mer were not considered). A variant was defined to alter a microRNA binding site if a putative binding site was present in either the REF or the ALT sequence, but not in both, or if the site class was different between the REF and the ALT sequences. Moreover, altering variants were classified as creating (destroying) a binding site if only the ALT (REF) sequence contained a binding site or if the ALT (REF) sequence contained a stronger binding site than the REF (ALT), according to the hierarchy 8mer > 7mer-m8 > 7mer-A1 match. Only microRNA families conserved across mammals or broadly conserved across vertebrates and expressed in lymphoblastoid cells were considered. Following Lappalainen et al. (2013), each microRNA was considered expressed if its expression value was >0 in at least 50% of the samples, and each microRNA family was considered expressed if at least one of its microRNAs was expressed.

**RBP motifs.** The candidate variants were all the intragenic SNPs. FIMO (Grant et al., 2011) was used to scan the REF and ALT sequences around each candidate variant, using as background the nucleotide frequencies on the sequence of all the analyzed genes. A motif was considered altered if its score was >80% the score of the perfect match in only one of two alleles. As in the case of microRNAs, only motifs corresponding to RBPs expressed in lymphoblastoid cell lines were considered. Enrichment was evaluated both for SNPs altering any RBP motif and for each expressed RBP separately.

#### GWAS Hits

We considered only the GWAS catalog records referring to a single genetic variant on autosomal chromosomes for which all the fields CHR_ID, CHR_POS, SNPS, MERGED, SNP_ID_CURRENT, and MAPPED_TRAIT_URI were available, as well as the RSID. The coordinates of the selected genetic variants in hg19 were derived from dbSNP Build 151. We thus obtained 56,672 genetic variants associated with at least one complex trait. Furthermore, starting from the EFO URI(s) reported for each association, we obtained the corresponding EFO Parent URI(s) from the EFO annotation file.

All variants examined as potential apaQTLs were considered as our candidate variants. A binary feature value of 1 was attributed to each candidate variant surviving LD pruning and associated to a trait, or with a tagging variant associated to a trait, as in the case of motif-altering variants. Enrichment was evaluated for all trait-associated variants together, for each single trait, and for trait categories defined based on the EFO ontology. Only traits and trait categories associated with at least 100 GWAS hits were analyzed. The same analysis was also performed after excluding all variants within the HLA locus, as defined by The Genome Reference Consortium (https://www.ncbi.nlm.nih.gov/grc/human/regions/MHC?asm=GRCh37).

### The rs10954213 Variant in SLE Patients

In the analysis of SLE patient RNA-seq data, we were interested in the *IRF5* gene only. Therefore, RNA-Seq reads were aligned to a reduced genome comprising the gene sequence and an additional 50 bp at its 3′ end using Bowtie v2.2.3 (Langmead et al., 2009) and TopHat v2.0.12 (Trapnell et al., 2009). As genotypic data were not available for these individuals, we inferred the rs10954213 variant status from the relative proportion of A and G in the RNA-Seq reads. Initially, individuals were considered homozygous for the reference (G) or for the alternative (A) allele when the same nucleotide was present in all the reads, and a single read with a different nucleotide was considered sufficient to call a heterozygous individual. Then, genotype quality was assessed using VCFx version 1.2b (Castelli et al., 2015; Lima et al., 2016) with default parameters to filter out low-confidence genotypes. In this way, we obtained 11 homozygotes for the reference allele, 22 heterozygotes, and 14 homozygotes for the alternative allele (**Figure S9**), while 52 individuals with missing genotype information were excluded from the subsequent analysis. Notably, the genotypes are in Hardy–Weinberg equilibrium (chi-squared *P* = 0.705). A Kruskal–Wallis test was then used to evaluate the differences in *m*/*M* values between genotypes.

## Data Availability

Publicly available datasets were analyzed in this study. This data can be found here: https://www.ebi.ac.uk/Tools/geuvadis-das/

## Author Contributions

EM: conceived and planned the work, performed most computational analyses, wrote the manuscript, approved the final version; FM: performed computational analysis, approved the final version; EG: performed computational analysis, approved the final version; SG: performed computational analysis, approved the final version; PP: conceived and planned the work, wrote the manuscript, approved the final version.

## Funding

This work was supported by a grant from Compagnia di San Paolo, Turin, Italy [grant Torino_call_L2_252].

## Conflict of Interest Statement

The authors declare that the research was conducted in the absence of any commercial or financial relationships that could be construed as a potential conflict of interest.
