# Supplementary material for: The Length of the Expressed 3′ UTR Is an Intermediate Molecular Phenotype Linking Genetic Variants to Complex Diseases
Source: Front Genet. 2019 Aug 16;10:714. doi: 10.3389/fgene.2019.00714 (PMC6707137; doi:10.3389/fgene.2019.00714)
Supplement: Supplementary file 1 [file DataSheet_1.pdf]

# Supplementary Material

## 1 SUPPLEMENTARY DATA

**File S2.** Significant apaQTL models. Table listing the significant models obtained in the apaQTL mapping analysis. For each model we report: the genetic variant identifier according to dpSNP137, as provided into the GEUVADIS dataset (SNP_ID), the NCBI Entrez ID (GENE_ID), the regression coefficient (BETA), the nominal P-value (NOMINAL_PVALUE), the corresponding empirical P-value (EMPIRICAL_PVALUE) and Benjamini-Hochberg corrected empirical P-value (FDR). In addition, multiple files listing the results of all the models that were fitted in each chromosome are publicly available on Mendeley Data (`http://dx.doi.org/10.17632/6d8w2p9bzf.1`).

**File S3.** Enrichment of trait-specific GWAS hits among apaQTL. The table includes the following information for each GWAS trait for which we found a significant enrichment: the URI of the trait in the EFO database (EFO_URI), the associated name (EFO_TERM) and its parent term in the EFO database (EFO_PARENT_TERM), the odds ratio (OR), its 95% confidence interval (95% CI) and the corresponding P-value before and after multiple testing correction by the Benjamini-Hochberg method (P-VALUE and FDR).

**File S4.** Enrichment of trait-specific GWAS hits among apaQTL, after the exclusion of genetic variants within the HLA locus. The table includes the following information for each GWAS trait for which we found a significant enrichment after the exclusion of genetic variants within the HLA locus: the URI of the trait in the EFO database (EFO_URI), the associated name (EFO_TERM) and its parent term in the EFO database (EFO_PARENT_TERM), the odds ratio (OR), its 95% confidence interval (95% CI) and the corresponding P-value before and after multiple testing correction by the Benjamini-Hochberg method (P-VALUE and FDR).

**File S5.** Annotation of gene structures. GTF file with the custom gene annotation that was used for all the analyses.

**File S6.** GTF file used for the computation of m/M values. For each gene the coordinates of the PRE and POST segments were obtained combining its structure annotation with the poly(A) sites reported by PolyADB_2. In addition the length of each of these segments is reported.

# 2 SUPPLEMENTARY TABLES AND FIGURES

## 2.1 Figures

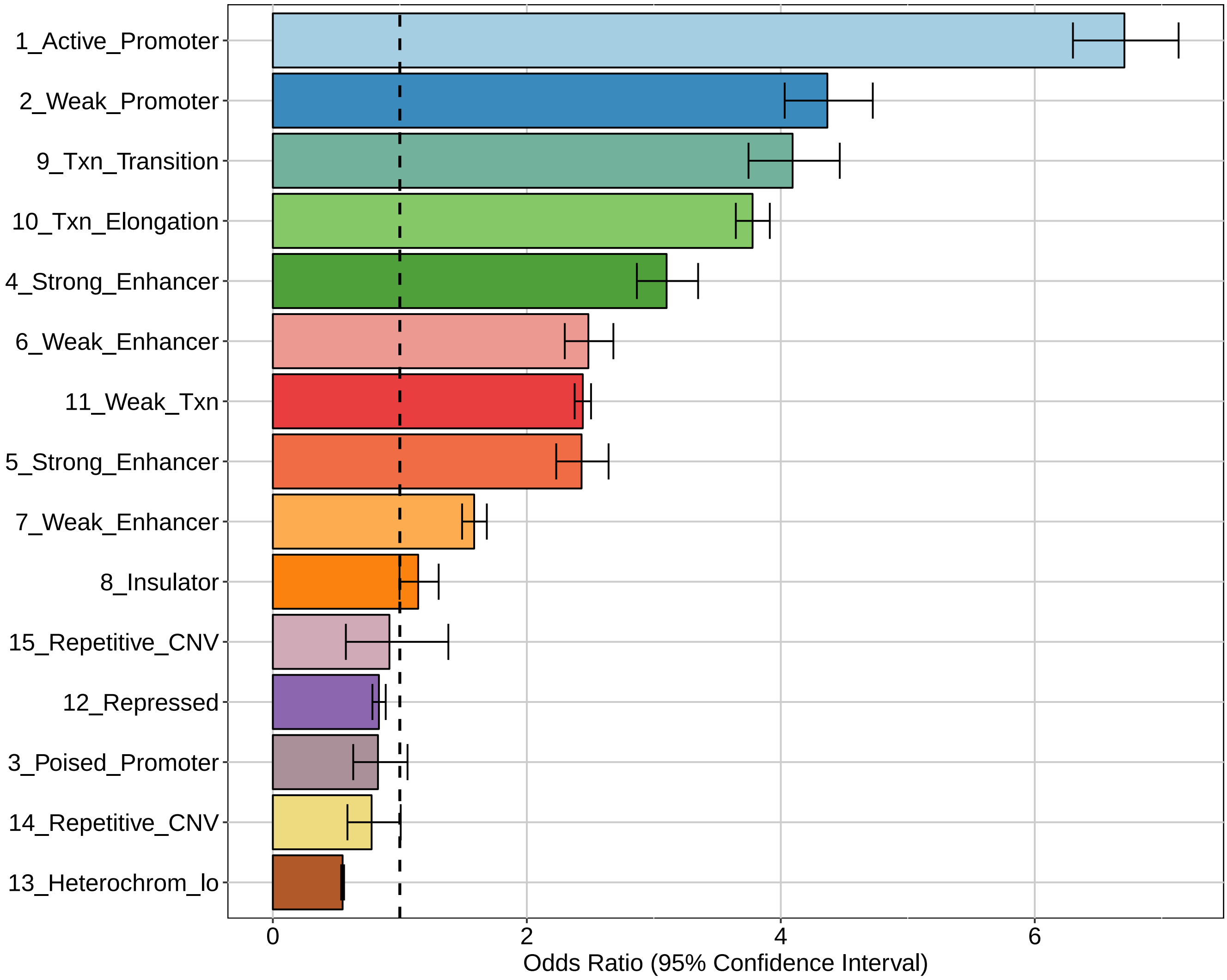


**Figure S1.** Enrichment of apaQTLs within chromatin states, taking into account all the 15 chromatin states reported in the ChromHMM annotation. For each of them, the OR obtained by logistic regression and its 95% CI are shown.

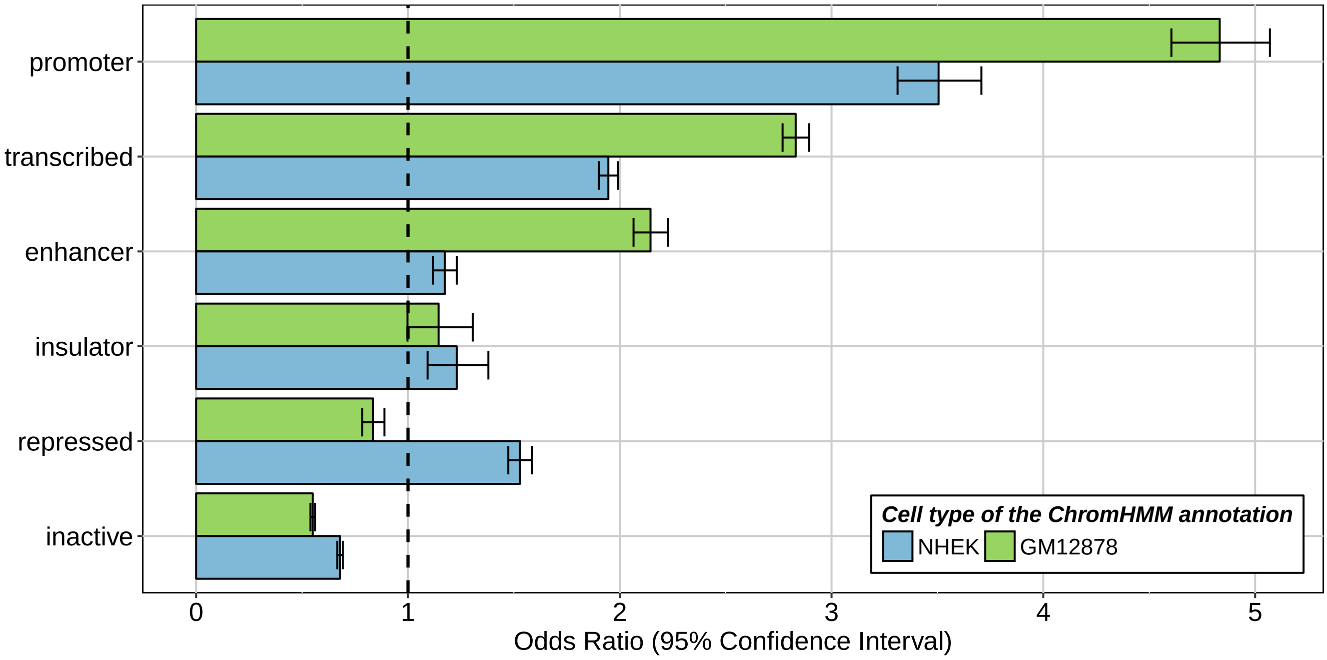


**Figure S2.** The results of the enrichment analysis performed with the broad chromatin states of the relevant cell type were compared with those obtained using the ChromHMM annotation of another cell type (NHEK). For each category, the OR obtained by logistic regression and the corresponding 95% CI are shown.

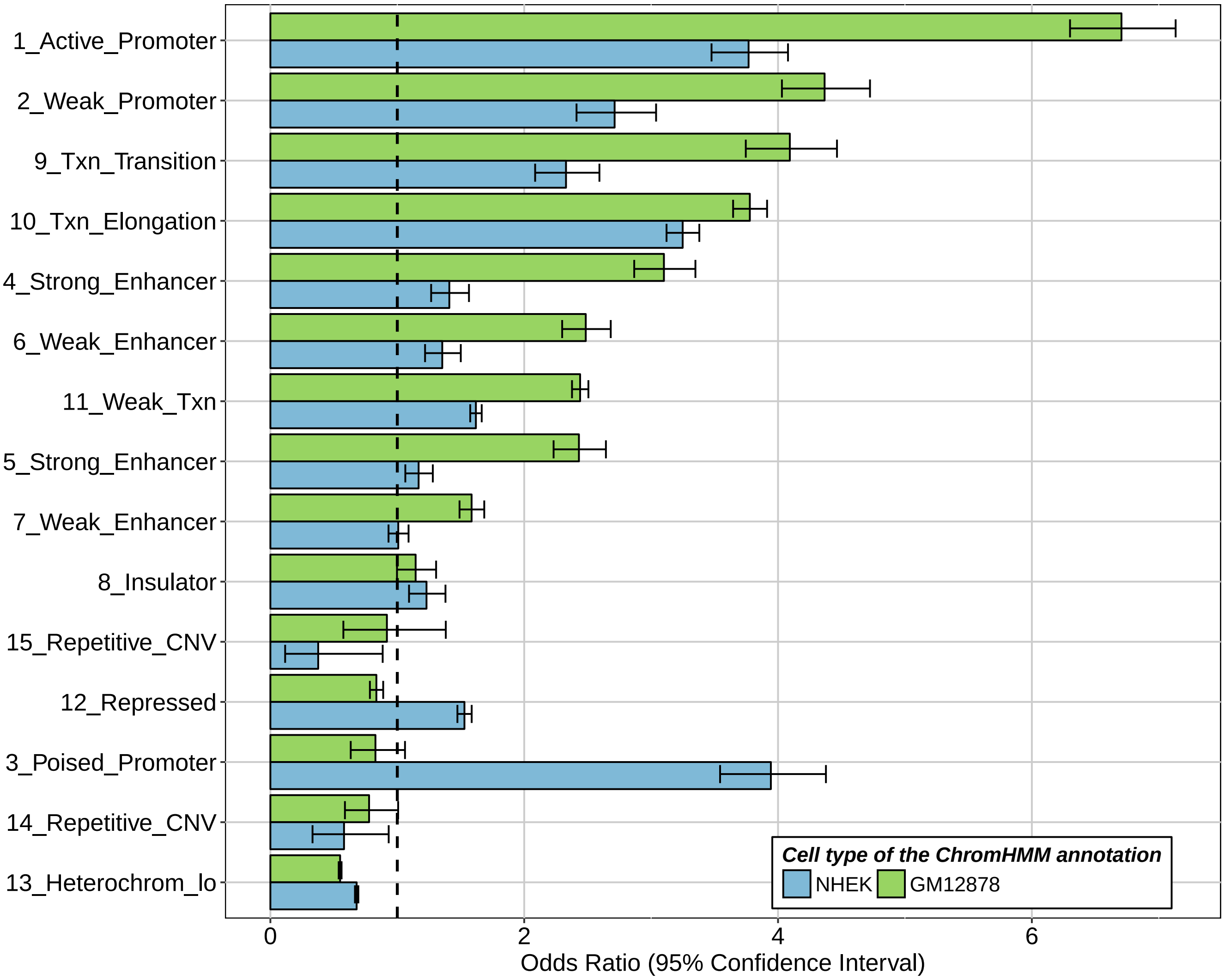


**Figure S3.** The results of enrichment analysis done performed with all the chromatin states of the relevant cell type were compared with those obtained using the ChromHMM annotation of another cell type (NHEK). For each category, the OR obtained by logistic regression and the corresponding 95% CIs are shown.

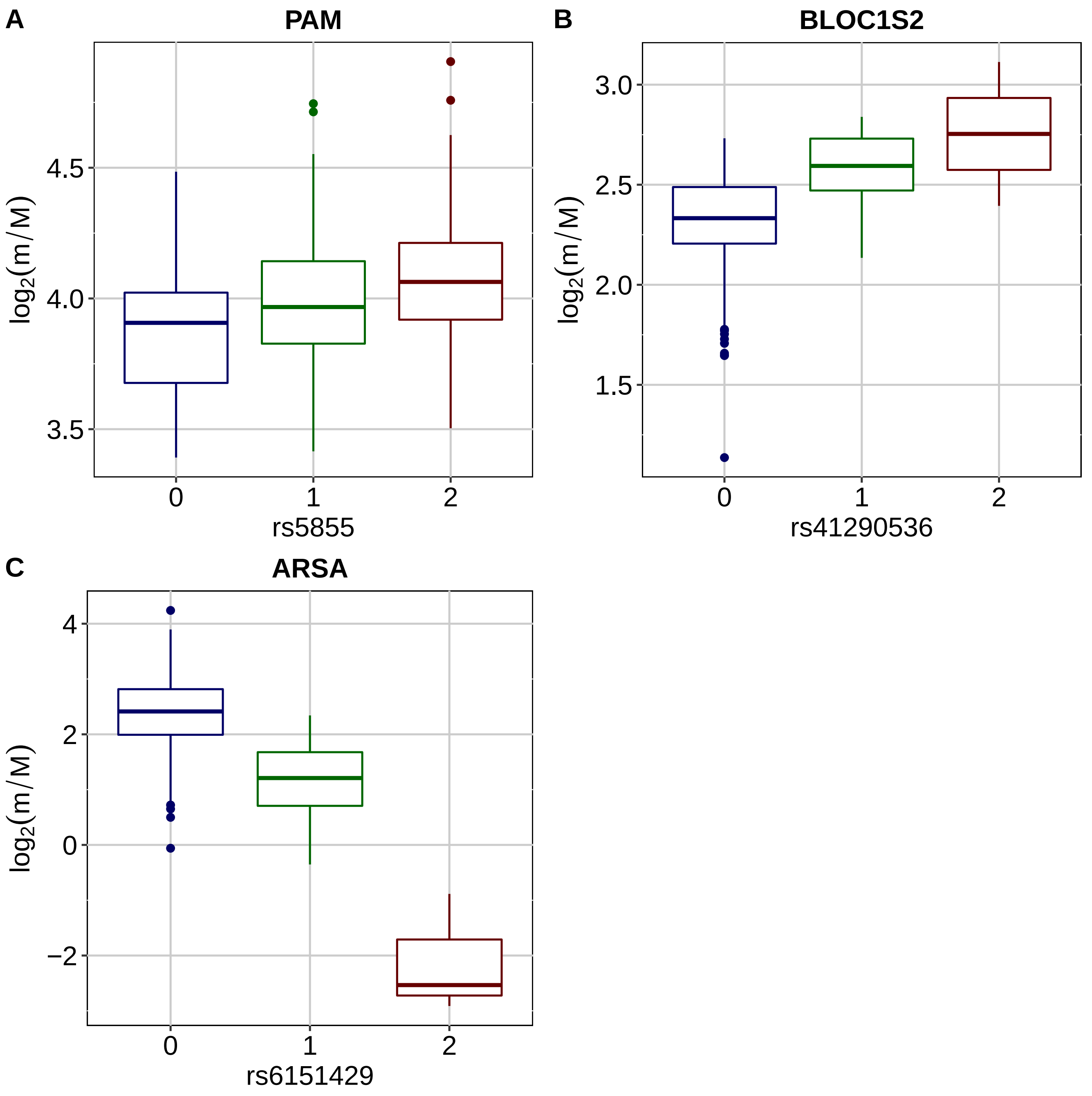


**Figure S4.** Boxplots showing the variation of the log2-transformed m/M values obtained for *PAM* (A), *BLOC1S2* (B) and *ARSA* (C), as a function of the genotype of the individuals for a single genetic variant that falls within the cis-window of the tested gene (rs5855, rs41290536 and rs6151429, respectively).

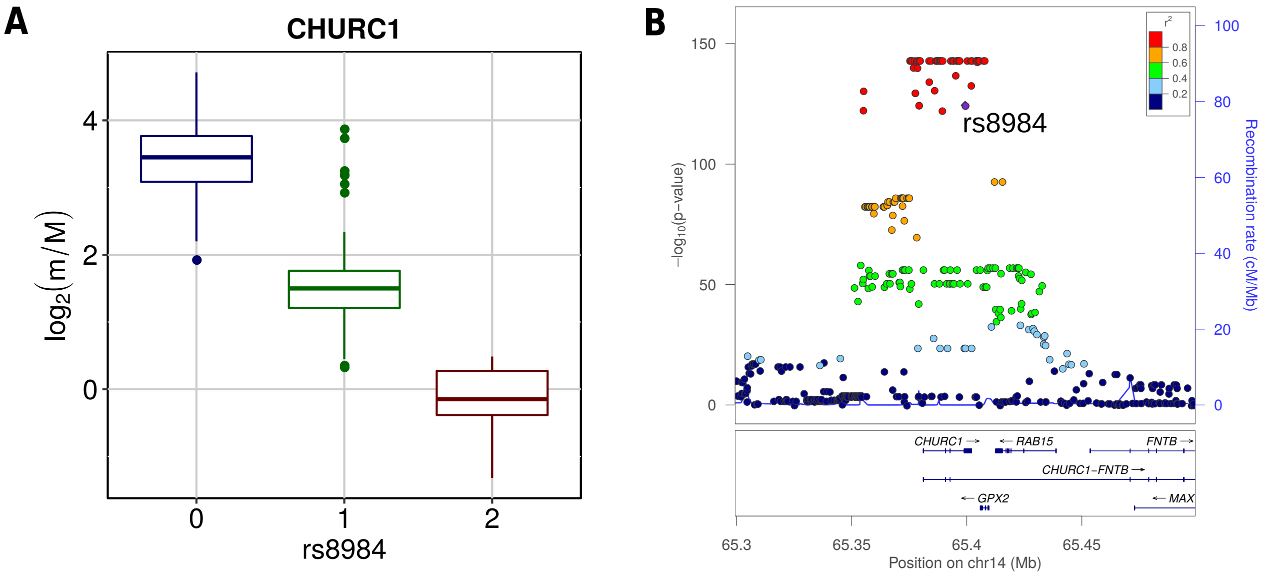


**Figure S5.** (A) Boxplot showing the variation of log2-transformed m/M values obtained for *CHURC1* as a function of the genotype of the individuals for rs8984. (B) LocusZoom plot illustrating the results obtained for *CHURC1* in the genomic region around rs8984 (100kb both upstream and downstream its genomic location).

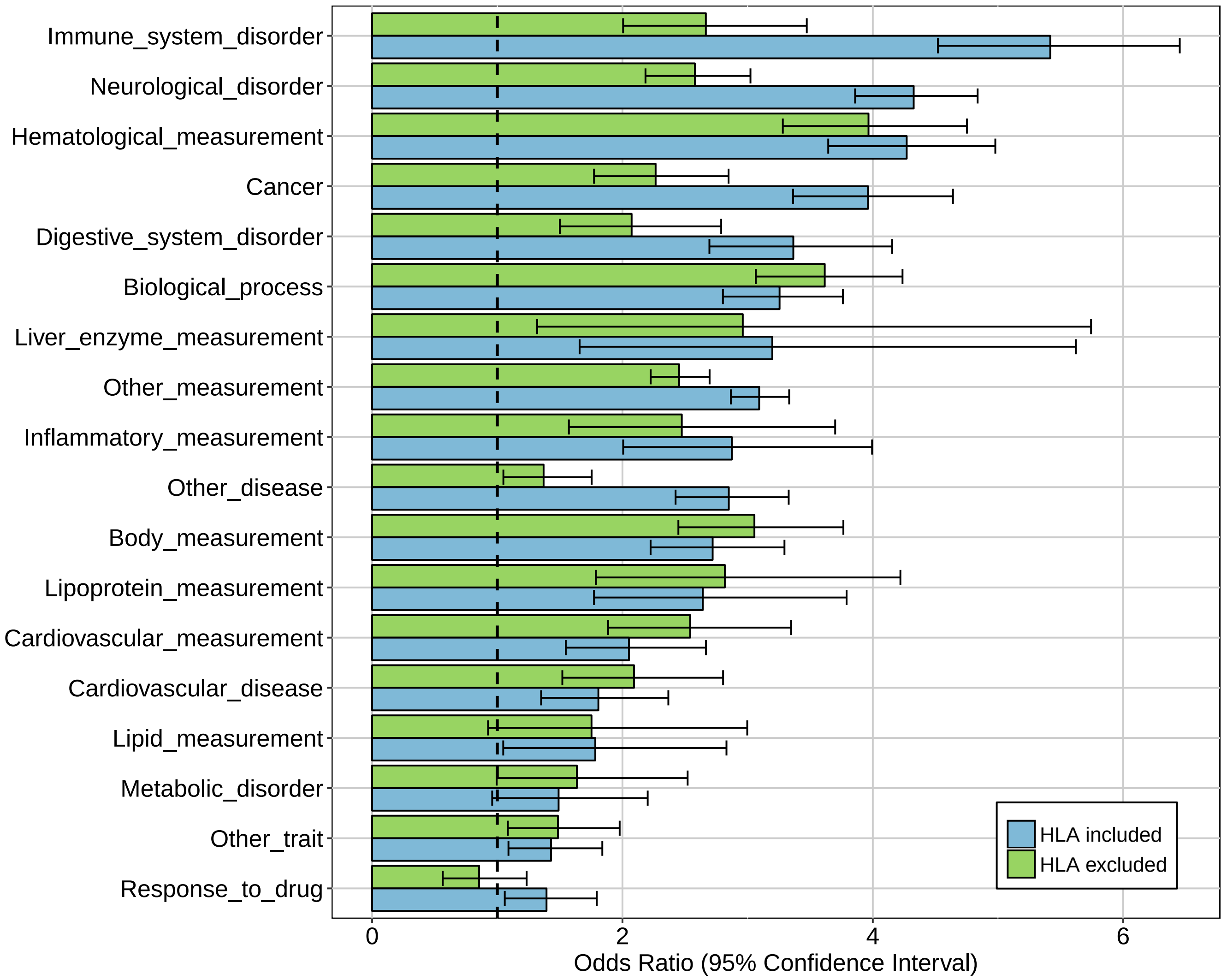


**Figure S6.** Comparison of the results of the enrichment analyses performed for multiple categories of complex traits considering all the studied genetic variants (HLA included) or after having excluded those that are located within the HLA locus (HLA excluded). For each category, the OR obtained by logistic regression and the corresponding 95% CIs are shown.

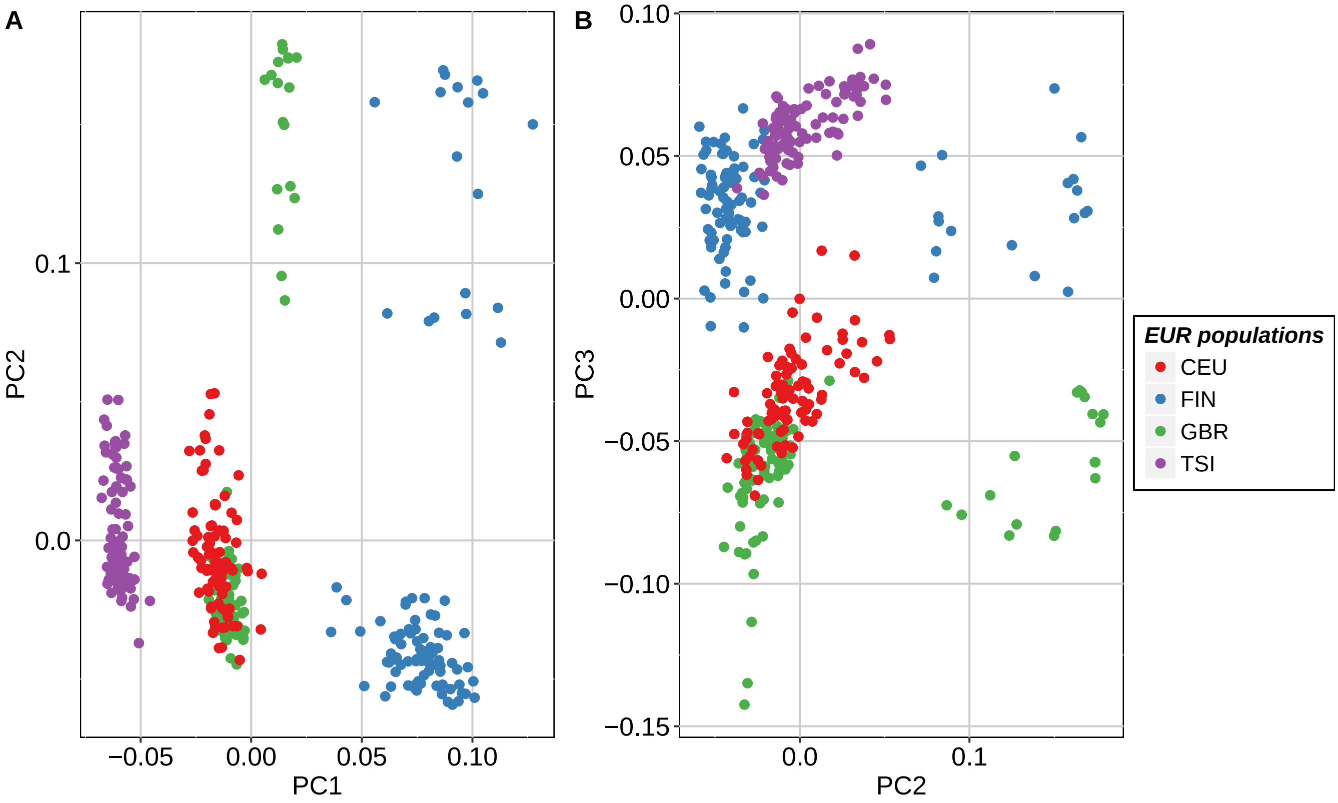


**Figure S7.** Principal Component Analysis (PCA) on the genotypic data of EUR individuals. Points are colored according to the subpopulation of origin: Utah Residents (CEPH) with Northern and Western European Ancestry (CEU), Finnish in Finland (FIN), British in England and Scotland (GBR) and Toscani in Italia (TSI).

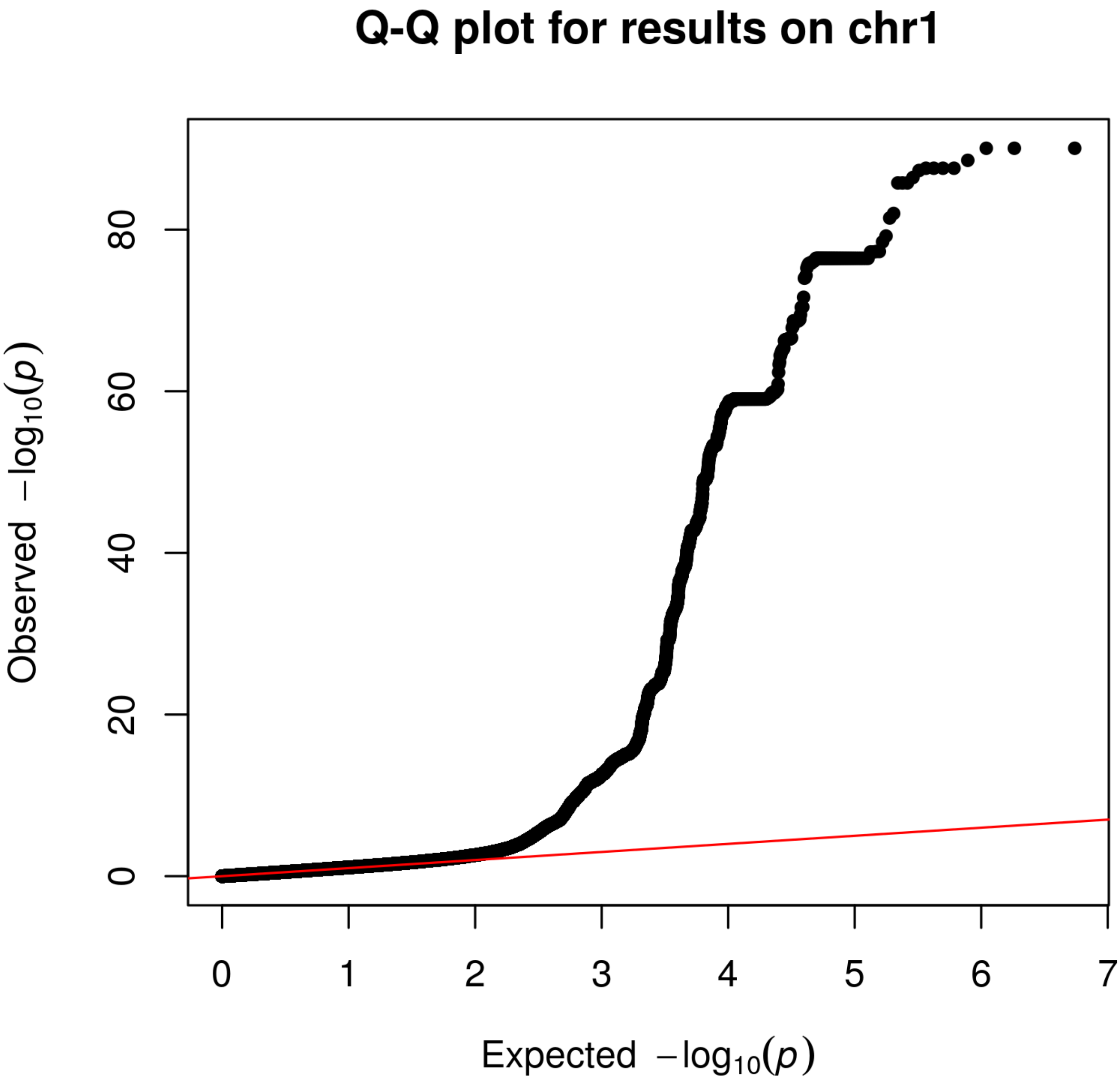


**Figure S8.** Q-Q plot comparing the distribution of P-values obtained fitting apaQTL models for genes on chr1 with the expected uniform distribution. It was generated by the CRAN R package `qqman`.

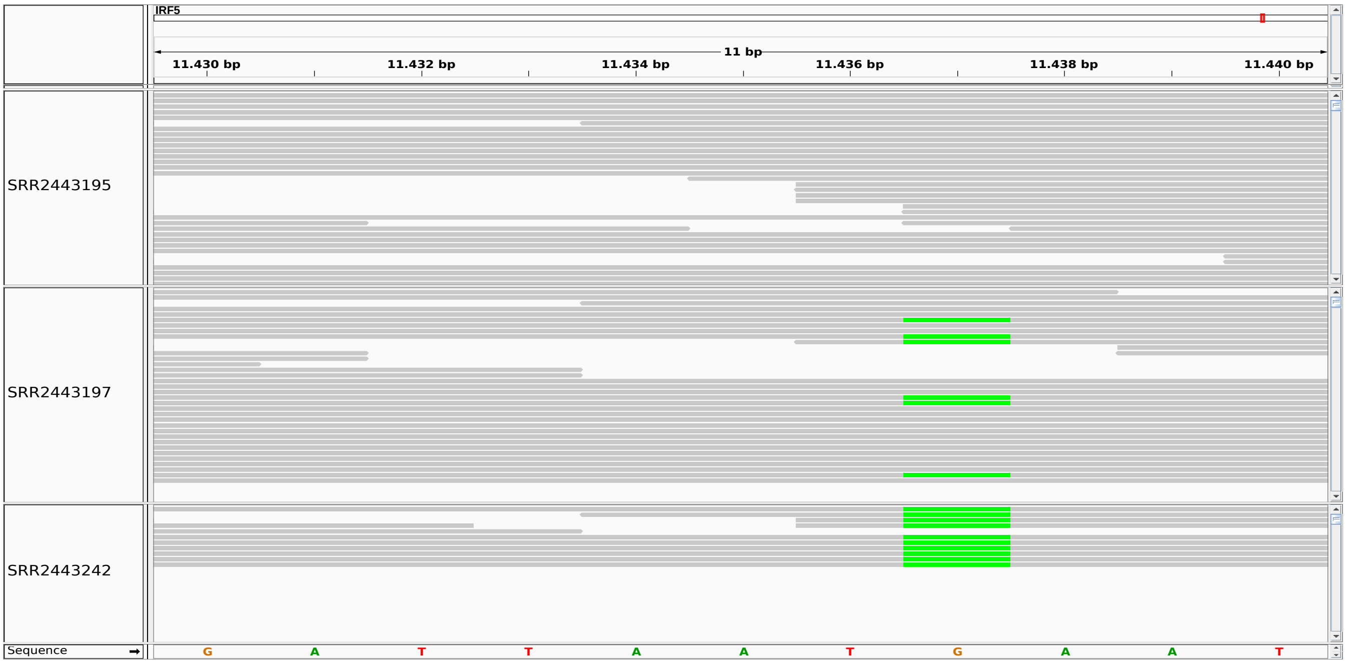


**Figure S9.** Genotypic information was not available for the SLE patients, therefore their genotype in correspondence of the rs10954213 genetic variant was inferred from RNA-Seq data. The figure shows the alignment of RNA-Seq reads in a region around the variant with respect to a reduced genome including only the *IRF5* gene and was generated using the Integrative Genomics Viewer (IGV) software. For example, the SRR2443195 individual (top panel) was considered homozygous for the reference allele, the SRR2443197 individual (central panel) was considered heterozygous and finally the SRR2443242 individual (bottom panel) was considered homozygous for the alternative allele.

## 2.2 Tables

**Table S1.** Enrichment of RBP-altering SNPs among intragenic apaQTL

| MOTIF ID | RBP NAMES | OR | 95% CI | P-VALUE | FDR |
|---|---|---|---|---|---|
| M016_0.6 | FMR1 | 3.72 | 1.01-11.3 | 0.0277 | 0.265 |
| M025_0.6 | HNRNPC | 1.77 | 1.06-2.83 | 0.0213 | 0.264 |
| M070_0.6 | ENSG00000180771;SRSF2 | 3.83 | 1.55-8.7 | 0.00196 | 0.0518 |
| M075_0.6 | TIA1 | 1.81 | 0.993-3.1 | 0.0389 | 0.326 |
| M081_0.6 | CSDA;YB-1 | 3.9 | 1.03-12.6 | 0.0284 | 0.265 |
| M089_0.6 | HNRNPL | 3.4 | 1.08-9.12 | 0.0216 | 0.264 |
| M122_0.6 | MEX3B;MEX3C;MEX3D | 3.85 | 1.03-12 | 0.0265 | 0.265 |
| M140_0.6 | ENOX1;ENOX2 | 3.16 | 1.36-6.76 | 0.00431 | 0.078 |
| M145_0.6 | RBM5 | 4.97 | 1.51-14.6 | 0.00441 | 0.078 |
| M147_0.6 | CNOT4 | 2.11 | 0.948-4.25 | 0.0478 | 0.362 |
| M156_0.6 | TIA1 | 1.81 | 1.05-2.96 | 0.0247 | 0.265 |
| M158_0.6 | HNRNPCL1 | 1.77 | 1.06-2.83 | 0.0213 | 0.264 |
| M160_0.6 | KHDRBS1 | 4.15 | 1.54-10.3 | 0.0027 | 0.0614 |
| M250_0.6 | CSDA | 2.93 | 0.939-7.73 | 0.041 | 0.326 |
| M256_0.6 | ACO1 | 1.92 | 1.07-3.25 | 0.0208 | 0.264 |
| M291_0.6 | EIF4B | 10.6 | 3.5-33.2 | 2.48e-05 | 0.00131 |
| M292_0.6 | EIF4B | 1.98 | 1.31-2.88 | 0.000643 | 0.0256 |
| M320_0.6 | MBNL1;MBNL2;MBNL3 | 1.66 | 1.2-2.25 | 0.00163 | 0.0518 |
| M333_0.6 | SRSF9 | 1.8 | 0.99-3.07 | 0.0398 | 0.326 |
| M344_0.6 | RBMX;RBMXL1;RBMXL2 | 1.77 | 1.4-2.2 | 6.38e-07 | 5.07e-05 |

**Table S2.** Enrichment of GWAS hits for different trait categories among apaQTL

| EFO URI | EFO TERM | OR | 95% CI | P-VALUE | FDR |
|---|---|---|---|---|---|
| EFO_0000540 | Immune_system_disorder | 5.42 | 4.52-6.45 | 1.99e-77 | 1.19e-76 |
| EFO_0000618 | Neurological_disorder | 4.33 | 3.86-4.84 | 1.89e-142 | 1.7e-141 |
| EFO_0004503 | Hematological_measurement | 4.27 | 3.64-4.98 | 2.43e-74 | 1.09e-73 |
| EFO_0000616 | Cancer | 3.96 | 3.36-4.64 | 3.72e-63 | 1.34e-62 |
| EFO_0000405 | Digestive_system_disorder | 3.37 | 2.69-4.15 | 4e-28 | 9e-28 |
| GO_0008150 | Biological_process | 3.26 | 2.8-3.76 | 8.22e-56 | 2.47e-55 |
| EFO_0004582 | Liver_enzyme_measurement | 3.2 | 1.66-5.62 | 0.000167 | 0.000215 |
| EFO_0001444 | Other_measurement | 3.09 | 2.87-3.33 | 8.45e-190 | 1.52e-188 |
| EFO_0004872 | Inflammatory_measurement | 2.87 | 2.01-3.99 | 1.71e-09 | 3.07e-09 |
| EFO_0000408 | Other_disease | 2.85 | 2.42-3.33 | 1.99e-38 | 5.11e-38 |
| EFO_0004324 | Body_measurement | 2.72 | 2.22-3.29 | 1.46e-23 | 2.92e-23 |
| EFO_0004732 | Lipoprotein_measurement | 2.64 | 1.77-3.79 | 4.98e-07 | 7.47e-07 |
| EFO_0004298 | Cardiovascular_measurement | 2.05 | 1.55-2.67 | 2.2e-07 | 3.61e-07 |
| EFO_0000319 | Cardiovascular_disease | 1.81 | 1.35-2.37 | 3.42e-05 | 4.73e-05 |
| EFO_0004529 | Lipid_measurement | 1.78 | 1.05-2.83 | 0.0218 | 0.0231 |
| EFO_0000589 | Metabolic_disorder | 1.49 | 0.959-2.2 | 0.0589 | 0.0589 |
| EFO_0000001 | Other_trait | 1.43 | 1.09-1.84 | 0.00741 | 0.00889 |
| GO_0042493 | Response_to_drug | 1.39 | 1.06-1.79 | 0.0135 | 0.0152 |

**Table S3.** Enrichment of GWAS hits for different trait categories among apaQTL, after the exclusion of genetic variants within the HLA locus

| EFO URI | EFO TERM | OR | 95% CI | P-VALUE | FDR |
|---|---|---|---|---|---|
| EFO_0004503 | Hematological_measurement | 3.97 | 3.28-4.75 | 3.16e-48 | 1.9e-47 |
| GO_0008150 | Biological_process | 3.62 | 3.06-4.24 | 1.27e-54 | 1.15e-53 |
| EFO_0004324 | Body_measurement | 3.05 | 2.45-3.76 | 2.8e-24 | 1.01e-23 |
| EFO_0004582 | Liver_enzyme_measurement | 2.96 | 1.32-5.74 | 0.00338 | 0.00468 |
| EFO_0004732 | Lipoprotein_measurement | 2.82 | 1.79-4.22 | 2.03e-06 | 3.94e-06 |
| EFO_0000540 | Immune_system_disorder | 2.67 | 2.01-3.47 | 2.32e-12 | 6.96e-12 |
| EFO_0000618 | Neurological_disorder | 2.58 | 2.18-3.02 | 2.9e-30 | 1.3e-29 |
| EFO_0004298 | Cardiovascular_measurement | 2.54 | 1.89-3.35 | 1.76e-10 | 3.95e-10 |
| EFO_0004872 | Inflammatory_measurement | 2.47 | 1.57-3.7 | 3.07e-05 | 4.61e-05 |
| EFO_0001444 | Other_measurement | 2.45 | 2.23-2.7 | 4.32e-75 | 7.78e-74 |
| EFO_0000616 | Cancer | 2.26 | 1.77-2.85 | 1.27e-11 | 3.26e-11 |
| EFO_0000319 | Cardiovascular_disease | 2.09 | 1.52-2.8 | 2.19e-06 | 3.94e-06 |
| EFO_0000405 | Digestive_system_disorder | 2.07 | 1.5-2.79 | 3.92e-06 | 6.41e-06 |
| EFO_0004529 | Lipid_measurement | 1.75 | 0.926-3 | 0.0587 | 0.0622 |
| EFO_0000589 | Metabolic_disorder | 1.64 | 0.995-2.52 | 0.0369 | 0.0415 |
| EFO_0000001 | Other_trait | 1.48 | 1.08-1.98 | 0.00981 | 0.0126 |
| EFO_0000408 | Other_disease | 1.37 | 1.05-1.75 | 0.0163 | 0.0196 |
| GO_0042493 | Response_to_drug | 0.855 | 0.564-1.23 | 0.43 | 0.43 |
